# Integration of LLMs and VLMs in plant stress phenotyping: From trait recognition to decision support

**DOI:** 10.1016/j.plaphe.2025.100161

**Published:** 2025-12-29

**Authors:** Elshan Musazade, Isack Ibrahim Mrisho, Jinshan Gao, Xianzhong Feng

**Affiliations:** a Key Laboratory of Soybean Molecular Design Breeding, Northeast Institute of Geography and Agroecology, Chinese Academy of Sciences, Changchun, 130102, PR China; b College of Chinese Medicinal Material, Jilin Agricultural University, Changchun, 130118, PR China

**Keywords:** Large language models, Vision-language models, Plant stress detection, Precision agriculture, Artificial intelligence in phenotyping

## Abstract

The integration of Large Language Models (LLMs) with Vision-Language Models (VLMs) holds transformative potential for plant stress phenotyping, enhancing high-throughput crop monitoring, trait identification, and decision support. Traditional phenotyping methods, often reliant on manual assessments and task-specific Machine Learning (ML) models, face persistent limitations in scalability, adaptability, and contextual interpretation, especially under complex and overlapping stress conditions. VLMs address these challenges by combining deep visual recognition with contextual reasoning, enabling real-time analysis of multimodal inputs such as high-resolution imagery, agronomic text data, and environmental sensor readings. Complementarily, LLMs contribute to text mining, semantic annotation of trait descriptors, and the integration of external knowledge via Retrieval-Augmented Generation (RAG), thereby enhancing the interpretability and adaptability of phenotyping workflows. This review critically evaluates the emerging role of integrating LLMs with VLMs in plant stress phenotyping, highlighting their applications in visual trait recognition, knowledge extraction, and autonomous decision-making. We synthesize current advances and identify key challenges, including data quality, domain-specific generalization, model transparency, and equitable access to AI technologies. As one of the first comprehensive reviews on this topic, we propose a forward-looking framework that integrates LLMs, VLMs, and RAG systems to enable scalable, explainable, and user-centric phenotyping solutions. This interdisciplinary convergence offers a promising pathway toward sustainable and resilient AI-driven agriculture.

## 1 Introduction

Ensuring global food security amidst accelerating climate change, resource constraints, and population growth demands urgent innovation in sustainable agriculture [1,2]. A major challenge in this regard is plant stress, which significantly constrains agricultural productivity and arises from both biotic and abiotic factors. Biotic stresses, including pests, pathogens, and weeds, cause yield losses of 17 %–40 %. In contrast, abiotic stresses such as drought, extreme temperatures, heavy metal toxicity, and salinity can reduce yields by up to 50 %, depending on crop species and regional conditions [3,4]. While the combined impact of these stressors is substantial, exact global estimates remain challenging due to overlapping and interactive effects.

Despite considerable progress in plant breeding and agronomic interventions, current approaches to plant stress phenotyping remain insufficient, particularly for rapid diagnosis and adaptive response to multiple, co-occurring stressors in large-scale agricultural systems. Traditional stress detection methods, such as expert field assessments and visual inspections, are inherently labor-intensive, subjective, and poorly scalable, limiting their suitability for modern precision agriculture [1,5]. Moreover, many stress-induced symptoms, such as leaf chlorosis, are nonspecific and overlapping across different stressors (e.g., drought vs nutrient deficiency), making accurate diagnosis in field conditions highly challenging [6]. Therefore, scalable, accurate, real-time stress monitoring systems are urgently needed to enable targeted interventions, mitigate yield losses, and enhance crop resilience.

Recent advances in Artificial Intelligence (AI), particularly Deep Learning (DL) and Large Language Models (LLMs), are opening unprecedented opportunities for automated and scalable plant stress management [[7], [8], [9]]. DL-based approaches, particularly Convolutional Neural Networks (CNNs), have demonstrated high accuracy in plant disease detection under controlled conditions, with some studies reporting results exceeding 99 % on curated datasets [10]. However, such performance is not universal and depends heavily on factors such as dataset quality, class balance, and the specific crop-disease context. Nevertheless, unimodal CNN models that rely exclusively on image data face significant challenges, primarily due to the need for large, high-quality annotated datasets, which are often lacking for many crops and stress conditions [11,12]. These constraints limit their generalizability and limit their applicability in real-world agricultural environments, which are inherently diverse, dynamic, and data-scarce.

A promising approach to bridging the expertise gap and enabling more flexible phenotype analysis lies in the integration of LLMs and Vision-Language Models (VLMs) [13,14]. LLMs, such as OpenAI's GPT series [15], Google's PaLM-E [16], and Meta's LLaMA [14], are capable of advanced semantic reasoning, few-shot learning (i.e., learning to perform tasks using only a few labeled examples), and cross-domain generalization from minimal supervision. VLMs are multimodal systems that combine an image encoder (e.g., CNNs, Vision Transformers (ViTs), or MAMBA-based encoders) with a text encoder (typically transformer-based), enabling joint alignment of visual and textual representations [17,18]. This design allows VLMs to perform cross-modal tasks such as image captioning, visual question answering, zero-shot classification, and image-based text generation [[19], [20], [21]]. Representative examples include CLIP, which aligns image-text embeddings at scale [22], and GroundingDINO, which enables open-set object detection through vision-language grounding [23]. In agricultural domains, VLMs facilitate real-time stress phenotyping by fusing high-resolution plant imagery with textual annotations and field records, offering enhanced interpretability and semantic trait recognition [24,25]. Their ability to generalize across stress conditions and crop types makes them especially valuable in data-limited settings. Furthermore, VLMs support automated dataset generation through captioning and annotation, streamlining phenotyping pipelines and reducing manual workload [13].

The integration of these large-scale AI systems represents a transformative leap in plant stress diagnostics. By combining visual recognition with natural language processing, integrated VLM-LLM frameworks can process heterogeneous data streams, such as drone imagery, sensor inputs (e.g., soil moisture and temperature), and farmer-generated text, enabling context-aware, adaptive, and real-time stress detection [[26], [27], [28], [29], [30]]. The few-shot learning capabilities of LLMs further support generalization across diverse phenotyping scenarios, even in the absence of large labeled datasets [31,32]. Additionally, their integration with remote sensing and proximal sensing platforms allows for localized and temporally synchronized decision-making in precision agriculture. Despite delays introduced by sources such as satellite imagery or manual observations, the fusion of multimodal data streams provides a comprehensive spatiotemporal understanding of plant health and stress responses, thereby improving the timeliness, precision, and scalability of phenotyping interventions [33].

Despite these advances, significant barriers hinder the widespread deployment of VLMs in agriculture. These include the scarcity of high-quality, multimodal datasets, high computational demands for training and deployment, and limited adaptability across languages and regions, which particularly affect smallholder and resource-limited farming systems [[34], [35], [36]]. To overcome these challenges, state-of-the-art Natural Language Processing (NLP) methods, such as Chain of Thought (CoT) prompting (guiding the model to reason step-by-step), Reinforcement Learning from Human Feedback (RLHF) (training the model using human preference signals), in-context learning (learning from examples provided in the prompt without updating model weights), and domain-specific instruction tuning (adapting the model using expert-written examples from a specific field), are being developed to enhance model alignment and reasoning in complex domains [37]. Nonetheless, further innovation is needed to adapt these models to agricultural-specific data and knowledge structures. Moreover, ethical and societal issues, including data privacy, bias, and equitable access, must be addressed to ensure the responsible adoption of AI in agriculture [38,39].

While VLMs and LLMs have gained significant attention in fields such as healthcare, robotics, and autonomous systems, their application to plant phenotyping, particularly under stress conditions, remains largely underexplored. To our knowledge, this is one of the first comprehensive reviews to examine how VLMs and LLMs, either independently or in synergy, can be applied across the whole plant stress phenotyping workflow, from visual trait recognition to intelligent decision support. This review critically evaluates the capabilities, limitations, and future directions of these models within the context of plant stress diagnostics. We highlight how LLMs can enhance vision-based systems through semantic reasoning, knowledge retrieval, and the creation of user-friendly, farmer-accessible interfaces. By establishing a conceptual and technical foundation, this work aims to support future research and responsible deployment of scalable, explainable AI frameworks in agricultural phenotyping.

## 2 Deep Learning, Large Language Models, and recent AI advances in plant stress detection

Machine Learning (ML) has emerged as a powerful tool in plant stress research, allowing for the analysis of complex datasets through various approaches, including supervised, unsupervised, and Reinforcement Learning (RL) [40]. Supervised learning involves training models with labeled data, such as Multilayer Perceptrons (MLPs), Naive Bayes (NB), Support Vector Machines (SVMs), and Random Forests (RF), to predict or classify plant stress levels. Unsupervised learning, in contrast, works with raw or unlabeled data, applying techniques such as k-means clustering, hierarchical clustering, and Principal Component Analysis (PCA) to discover underlying patterns and structures [41]. RL, which learns from interactions with the environment to maximize cumulative rewards, has been increasingly applied to plant phenomics [42], gene function prediction [43], and the identification of stress-responsive gene modules [44].

DL, a subset of ML, has become a leading approach in plant stress detection and classification due to its superior ability to automatically extract complex patterns from large datasets. Among DL methods, CNNs are particularly effective for image-based tasks, where they learn hierarchical features from raw pixel data. Various DL models, such as AlexNet and GoogleNet [45], Night-CNN [46], VGG16, VGG19, ResNet-50 [47], and InceptionV3 [48], have demonstrated high accuracy in plant disease detection. CNNs, including specialized models such as Night-CNN, have demonstrated exceptional performance in detecting diseases in crops such as potato (*S. tuberosum*), tomato (*Solanum lycopersicum*), and chili pepper (*Capsicum annuum*), achieving accuracy rates of 93 %–95 % [46]. Studies using ResNet-50 on the Plant-Village dataset reported an accuracy of 98.98 %, demonstrating the robustness of these models for real-world agricultural applications [47].

In recent years, ViTs have been increasingly recognized as a viable alternative to CNNs for image recognition tasks, exhibiting substantial improvements in both efficiency and accuracy. Introduced in 2021, ViTs decompose images into patches and train on these sequences, providing four times more computational efficiency than previous CNN models. Research on ViTs for plant disease detection, such as tomato leaf disease, has demonstrated accuracies ranging from 96.7 % to 99.7 % [48], making ViTs a promising tool for early plant disease detection.

LLMs represent a breakthrough in AI, integrating multiple ML strategies, including supervised, unsupervised, and RL. LLMs, such as Generative Pre-Trained Transformers (GPT), are unsupervised and pre-trained on massive textual datasets before undergoing supervised fine-tuning. These models perform reasoning, code generation, and decision-making [49,50]. LLMs also leverage self-supervised learning, generating supervisory signals from input data, which is valuable for biological data analysis where labeled datasets are scarce. For instance, ChatGPT has demonstrated the ability to analyze biological sequence data, aiding in plant stress research at the genetic level [51].

Building on the progress of both vision models and LLMs, VLMs provide an integrative framework that combines image encoders, typically CNNs or ViTs, with text encoders, often transformer-based language models, to enable joint visual-textual representation learning and cross-modal reasoning. VLMs have demonstrated strong performance in tasks such as image captioning, zero-shot classification, and visual question answering. Representative examples include CLIP (Contrastive Language-Image Pretraining), which learns aligned image-text embeddings for zero-shot recognition [22]; BLIP-2, which employs a lightweight Query Transformer to connect frozen vision encoders with LLMs for efficient multimodal pretraining [52]; and emerging hybrid frameworks such as YOLOPC + GPT-4, where YOLOPC provides lightweight pest and disease detection and GPT-4 generates domain-specific diagnostic reports, achieving both high detection accuracy and reasoning accuracy in agricultural applications [53].

**Fig. 1:**
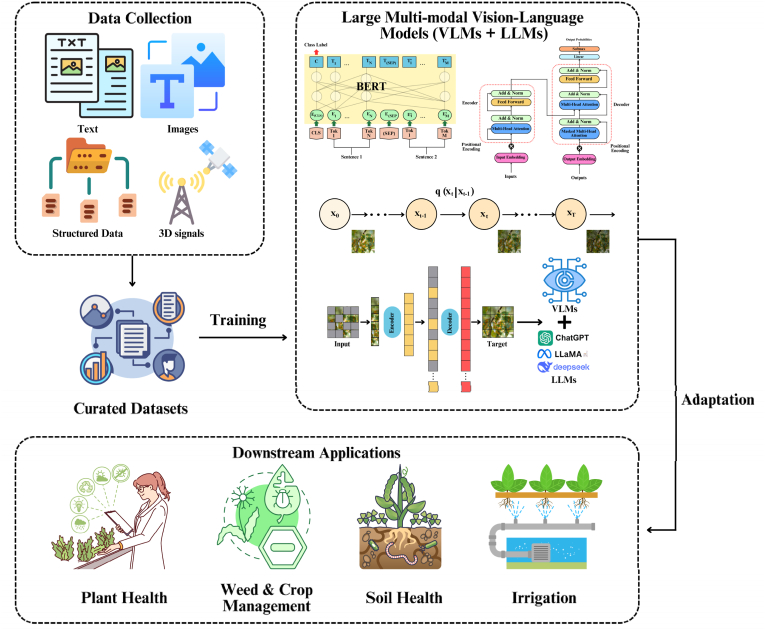
**Conceptual pipeline for developing large multimodal VLMs in agriculture.** The workflow begins with the collection of diverse multimodal data, including text, images, structured records, and 3D signals, which are processed into curated datasets for training. LLMs such as ChatGPT, LLaMA, and DeepSeek function as language backbones that, when integrated with vision encoders (e.g., CNNs, ViTs), form VLMs capable of joint visual-textual reasoning. While the figure highlights image-based workflows as a representative example, the framework is inherently multimodal and adaptable to additional data types. The resulting models support a range of downstream applications, including plant health monitoring, weed and crop management, soil health assessment, and precision irrigation, thereby enabling advanced, context-aware decision support in plant stress phenotyping and precision agriculture.

The integration of LLMs with vision-based AI models, such as CNNs and ViTs, marks a significant advancement in plant stress research. These hybrid approaches, commonly referred to as VLMs, are capable of analyzing visual and textual data simultaneously, thereby improving the depth, accuracy, and interpretability of stress detection. Unlike traditional ML methods, LLMs excel at processing complex and heterogeneous datasets, including multi-omics and environmental metadata. Classical ML algorithms typically require extensive data curation to minimize noise, whereas advanced DL models, including LLMs, can efficiently integrate and analyze diverse, high-dimensional biological data [51,54]. Their versatility enables fine-tuning for specialized tasks, such as gene function prediction and biomarker identification, providing nuanced insights into plant health and resilience. When combined with VLMs, these capabilities extend beyond trait recognition to support contextual interpretation and informed decision-making in plant stress phenotyping. Fig. 1 provides an overview of this integrative pipeline, from multimodal data collection and model development to downstream agricultural applications.

Building on these foundations, numerous AI-driven approaches have advanced the detection of plant stress. For instance, Dawei et al. (2019) developed a pest detection system utilizing transfer learning, achieving an accuracy of 93.84 % [55]. Wang et al. (2021) introduced ITC-Net, a model for recognizing vegetable diseases using image-text collaboration, achieving 99.48 % accuracy [56]. Similarly, Zhou et al. (2021) proposed ITK-Net, integrating image-text and knowledge graphs (KGs) (structured networks of facts linking entities and their relationships) for crop disease identification, achieving 99.63 % accuracy, 99 % precision, 99.07 % sensitivity, and 99.78 % specificity [6]. Additionally, Savarimuthu et al. (2021) conducted a comparative analysis of YOLOv4, EfficientDet, and Scaled-YOLOv4 for early plant disease detection, identifying Scaled-YOLOv4 as the most effective model [57].

Abbas et al. (2021) utilized C-GANs to generate synthetic tomato leaf images and employed DenseNet121 for classification, achieving an accuracy of up to 99.51 % and outperforming existing methods [58]. Feng et al. (2021) combined hyperspectral imaging with deep transfer learning for rice (*Oryza sativa*) disease detection across various varieties, achieving an accuracy of over 88 % [59]. Zhang et al. (2022) introduced Multi-ResNet34, a multimodal fusion model that combines tomato disease images and environmental data, achieving 98.9 % accuracy in diagnosing six tomato diseases and outperforming single-modal models [60]. Wang et al. (2022) developed an optimized YOLOv5 for real-time detection, achieving a 11.8 % improvement in runtime and a 3.98 % increase in accuracy, with an F1 score of 92.65 % [61]. T. Wang et al. (2022) proposed MPest-RCNN, achieving 99.11 % accuracy in pest detection [62]. Zhao et al. (2022) designed a CNN with Convolutional Block Attention Module (CBAM) for potato late blight detection, achieving an accuracy of 99.43 % [63].

Shin et al. (2023) applied Near-Infrared Spectroscopy (NIS) combined with Artificial Neural Networks (ANNs) for early detection of potato *Verticillium wilt*, achieving up to 94 % accuracy in training and 84 % accuracy in deployment, enabling detection as early as two days post-inoculation before visible symptoms [64]. Qadri et al. (2023) employed YOLOv8 to rapidly detect and segment sweet orange leaf diseases, achieving over 99 % precision, recall, and a mean average precision (mAP), demonstrating its high potential for agricultural disease management [65]. Wang et al. (2023) employed an enhanced Swin Transformer for cucumber (*Cucumis sativus*) leaf disease, achieving an accuracy of 98.97 % [66]. Sankareshwaran et al. (2023) proposed CAHA-AXRNet, a hummingbird algorithm-based RetinaNet, for rice disease detection with an accuracy of 98.1 % [67]. Reis and Turk (2024) presented MDSCIRNet, a transformer-based model for potato disease detection, achieving 99.24 % accuracy [68]. Malik and Mahmud (2024) applied YOLOv9 for weed detection using two open-source datasets, achieving a mAP of 94 % and 85 %, respectively, and outperforming YOLOv8 by 2.15 % in accuracy, while also achieving superior inference speed [69]. Zhang and Lv (2024) introduced TinySegformer, a lightweight transformer-based segmentation model for agricultural pest detection, achieving high accuracy (93 %) and real-time processing (32.7 FPS) on edge devices, outperforming models such as UNet and DeepLab [70].

Furthermore, Kumar et al. (2024) highlighted the potential of LLMs in disease prevention and control by analyzing disease transmission pathways and environmental factors and recommending interventions [39]. Yang et al. (2024) emphasized the use of LLMs for real-time control measures and pesticide recommendations [71]. Tzachor et al. (2023) and Ting et al. (2023) proposed LLM-based agricultural advisory systems (LLM-driven question-answering) using NLP for dynamic knowledge delivery [72,73].

Collectively, these advances in AI-driven plant stress detection, including DL, multimodal fusion, and transformer-based architectures, provide a robust foundation for developing sophisticated LLM-based VLMs. However, while several studies report near-perfect accuracies of 98–99 % and above, these figures should be interpreted with caution. In most cases, such results are achieved on relatively small or curated datasets under controlled conditions, where variability in lighting, environment, and genotype diversity is limited. When deployed in real-world field scenarios, model performance often declines due to domain shifts, co-occurring stresses, and noisy inputs, meaning that reported benchmarks may overstate robustness and generalizability. Thus, despite their high performance in isolated tasks, current models often lack integrated multimodal reasoning and language-based decision support, underscoring both the limitations of existing approaches and the growing need for LLM-powered vision-language systems to address real-world agricultural challenges.

These findings highlight not only the progress of AI in plant stress detection but also the emerging role of LLMs in agricultural diagnostics and advisory systems. Recent advances illustrate a clear evolution from traditional ML and CNN-based models toward transformers, multimodal fusion, and early LLM applications. Conventional DL approaches (e.g., CNNs, YOLO variants, and ViTs) have achieved high accuracy across diverse crops but remain constrained by dependence on large annotated datasets and limited interpretability. Multimodal strategies, such as integrating image, text, and environmental data or augmenting limited datasets with synthetic imagery, represent important transitional steps that foreshadow the reasoning capabilities of VLMs. Emerging studies now demonstrate the added value of LLMs, which can analyze disease transmission pathways, recommend control measures, and provide interactive advisory systems through question answering. Taken together, these developments illustrate a trajectory from perception-focused vision models toward integrated LLM/VLM frameworks that combine accurate detection with contextual reasoning and decision support.

## 3 Advantages of LLMs over conventional AI approaches in agricultural applications

LLMs have consistently demonstrated superior performance compared to conventional DL and ML approaches across a range of agricultural applications. Their advantages are particularly evident in tasks requiring contextual understanding, semantic reasoning, and real-time decision-making. For example, the domain-specific pre-trained agricultural Bidirectional Encoder Representations from Transformers (BERT) model outperformed standard transformer-based architectures in text classification tasks, achieving higher macro-F1 scores and overall accuracy [34]. In Named Entity Recognition (NER), LLMs such as GPT-4 and Multilingual BERT exhibited enhanced adaptability and precision when extracting agricultural entities from unstructured data, a task where conventional models often falter [74]. Furthermore, in diagnostic settings, LLMs have improved the efficiency of real-time agricultural information retrieval while reducing computational costs by shifting the logical reasoning component to GPT-4 rather than embedding it in end-to-end multimodal networks, thereby enabling scalable deployment on resource-constrained agricultural devices [53].

Beyond performance metrics, LLMs offer several functional advantages that position them as transformative tools in modern agriculture. Notably, their capacity to process complex, context-dependent tasks, such as semantic matching in food-nutrition systems or adaptive decision-making in precision farming, extends far beyond the capabilities of traditional ML and DL frameworks [75,76]. Their ability to generate accurate, context-aware outputs is especially beneficial in domains such as agricultural entity extraction, where domain-specific terminology poses challenges for non-specialized models [77]. Importantly, LLMs support zero-shot learning (i.e., performing tasks without task-specific training examples), enabling them to perform well even when labeled data is scarce, a common limitation in agricultural datasets [78].

The integration of LLMs with other AI technologies further amplifies their utility. For instance, combining GPT-4 with YOLOPC has produced lightweight yet high-accuracy diagnostic frameworks that significantly reduce parameter counts without compromising detection performance [53]. Rather than directly processing images, LLMs function as reasoning modules within multimodal pipelines, where outputs from vision encoders (e.g., CNNs, ViTs) can be synthesized with satellite imagery, sensor data, and textual field reports to generate unified, context-aware predictive insights. This multimodal integration extends beyond the capabilities of conventional DL architectures, enhancing the reliability of stress detection and management systems in agriculture and enabling more comprehensive, data-rich decision support [79].

In summary, the contextual reasoning, transferability, and multimodal capabilities of LLMs not only address key limitations of conventional ML/DL approaches but also lay the foundation for next-generation intelligent agricultural systems. These attributes make LLMs indispensable in advancing real-time, accurate, and scalable solutions for plant stress management and precision farming.

## 4 Current applications of LLMs in plant stress management

### 4.1 Image identification and classification

The integration of DL and large multimodal VLMs is revolutionizing agricultural diagnostics by enhancing the accuracy, efficiency, and scalability of plant stress identification and classification. With the increasing complexity of agricultural challenges, particularly in disease and pest management, the fusion of image and language-based AI models offers a promising pathway to precision agriculture [79]. Recent studies highlight significant advances in lightweight detection frameworks, mobile AI applications, multimodal benchmarks, and transformer-based architectures, each contributing to more explainable and actionable solutions for plant stress management [53,80,81].

Focusing on multimodal disease detection, Cao et al. (2023) introduced a recognition method called Image-Text-Label Multimodal Language Pretraining (ITLMLP), which integrates image-text multimodal contrastive learning, image self-supervised contrastive learning, and label-guided distance measurement in a shared feature space. This model was trained on a dataset from the Image Database of Agricultural Diseases and Pests (IDADP), comprising 3200 high-quality field images covering four major cucumber diseases: downy mildew, anthracnose, powdery mildew, and target spot (800 images per class). The proposed ITLMLP achieved a recognition accuracy of 94.84 %, demonstrating the capacity of VLMs to leverage multimodal cues for improved plant disease diagnostics [80]. Complementing this, Qing et al. (2023) proposed YOLOPC, a lightweight yet high-performing pest and disease detection model tailored for citrus crops. YOLOPC achieved 94.5 % accuracy while using 75 % fewer parameters than YOLOv5-nano, making it particularly suitable for deployment on embedded agricultural devices. The model was trained on a dataset comprising 1266 high-resolution RGB images, which included 544 images of adult citrus psyllids (*Diaphorina citri*) on fruit trees and 722 images of citrus leaves affected by Huanglongbing (HLB), anthracnose, citrus canker, and nutrient deficiencies. To enhance field-level diagnostics, the classification outputs from YOLOPC were mapped into structured textual prompts through a custom image-to-text conversion pipeline. These prompts were then processed by GPT-4, which served as an induction and reasoning module to generate domain-specific diagnostic reports contextualizing the visual predictions. Expert evaluation showed that GPT-4 achieved 90 % reasoning accuracy in producing reports that matched expert-written diagnoses. This framework illustrates a practical mode of combining VLM components with LLMs: the vision model provides reliable disease recognition, while the LLM contextualizes outputs into interactive, expert-level reports for farmers. Notably, the study highlights how integration is not merely “combining outputs,” but involves a structured mapping process that bridges visual classification and natural-language reasoning [53].

Expanding AI solutions to mobile platforms, Madaan et al. (2023) developed a hybrid framework in which conventional DL models (MobileNetV2, ResNet50) achieved 97–99 % accuracy in early crop disease detection, while an integrated LLM module provided tailored recommendations for disease prevention, fertilizer selection, and pesticide alternatives. This combination highlights how LLMs extend beyond perception to deliver context-aware decision support, emphasizing scalability and Explainable AI (XAI) for rural deployment [81].

To benchmark AI capabilities in plant stress detection, Arshad et al. (2024) introduced AgEval, a comprehensive multimodal benchmark encompassing 12 distinct plant stress-related tasks across identification, classification, and quantification categories. The benchmark incorporates diverse open-access datasets that include RGB images of seeds, leaves, weeds, and insects, addressing multiple stress domains. For seed morphology identification, the Durum Wheat Dataset and Soybean Seeds Dataset capture stress-induced variations in seed characteristics. For foliar stress detection, datasets such as Mango Leaf Disease, Bean Leaf Lesions Classification, and Soybean Diseases provide visual data on biotic and abiotic stresses affecting leaves. For pest identification, the DeepWeeds and Dangerous Farm Insects datasets include images of stress-inducing weeds and harmful insects. Classification tasks utilize datasets such as YELLOW-RUST-19, Fusarium Wilt in Chickpea, and the IDC Soybean Dataset, which annotate disease severity and abiotic stress tolerance based on expert-defined labels. For quantification, the InsectCount Dataset enables the estimation of pest density, while PlantDoc supports the segmentation of disease areas to calculate affected leaf surface percentages [82].

Using this benchmark, Arshad et al. evaluated state-of-the-art LLMs/VLMs under zero- and few-shot settings. The Gemini-pro-1.5 model outperformed others in zero-shot learning (F1-score: 50.45 %), excelling in mango (*Mangifera indica*) leaf disease and insect identification tasks. GPT-4o achieved the highest few-shot performance (F1-score: 73.37 %), particularly in tasks involving durum wheat (*Triticum durum*) and bean leaf lesion detection. Meanwhile, Claude-3.5-sonnet led in classification and quantification tasks (Normalized Mean Absolute Error (NMAE): 12.78 %). A key insight from the study was that example relevance during prompt construction improved performance by 15.38 %, highlighting the critical role of curated and domain-specific datasets in enhancing the accuracy of multimodal LLMs for real-world agricultural stress scenarios [82].

Pushing the boundaries of transformer-based architectures, Lu et al. (2024) developed a multimodal transformer model that aligns image and text features through self-attention mechanisms, significantly outperforming conventional DL models (AlexNet, GoogLeNet, VGG, and ResNet). The model was trained on a diverse dataset that included images of healthy rice and rice affected by diseases such as blast disease and yellow leaf disease, collected from various growth stages. The dataset was sourced from the West District Botanical Garden of China Agricultural University, along with publicly available images from different regions and climatic conditions. Achieving 95 % precision, 92 % recall, and 94 % accuracy in disease classification, it also outperformed BLIP, mPLUG-Owl, InstructBLIP, and BLIP2 in image captioning, and surpassed SSD, RetinaNet, CenterNet, and YOLOv8 in object detection. Ablation studies confirmed that cross-modal integration was essential for optimal performance, highlighting the importance of synergizing visual and textual data for agricultural diagnostics [83].

In a similar effort, Hue et al. (2024) introduced a GPT-4-based multimodal system for plant disease diagnosis, which combines image recognition and NLP with a comprehensive Korean plant disease and pesticide registry, encompassing 1420 host plants, 37,467 pesticides, and 2462 pathogen cases. This system leverages structured molecular data in conjunction with AI-driven vision analysis, providing scalable and accurate pest management solutions while overcoming the traditional limitations of subjective visual assessments and labor-intensive molecular diagnostics [84].

Regarding real-time AI deployment in agriculture, Kumar et al. (2024) developed a hybrid framework for the detection and management of coffee (*Coffea arabica*) leaf diseases, combining YOLOv8 for real-time instance segmentation with GPT-3.5 for disease diagnosis and treatment recommendations. The system was trained on a diverse dataset sourced from Kaggle, open-source repositories, and field-collected images, targeting diseases such as Phoma and pests such as leaf miner (*Liriomyza sp.*). YOLOv8, pre-trained on the COCO dataset, was fine-tuned via transfer learning for coffee-specific segmentation tasks. To enhance the reliability of LLM outputs, the authors integrated a Retrieval-Augmented Generation (RAG) framework, enabling GPT-3.5 to access dynamic, domain-specific information and mitigate issues such as hallucination and reliance on static knowledge. This pipeline enhances not only diagnostic accuracy but also facilitates real-time monitoring and collaborative dataset expansion, underscoring the potential of adaptive, multimodal AI systems in field-ready plant disease phenotyping and sustainable crop management [39].

To address the challenge of data scarcity, Dhavale et al. (2024) combined Generative Adversarial Networks (GANs) for data augmentation with CNNs for high-precision crop disease classification. Furthermore, they incorporated Langchain Llama-based conversational AI, offering chatbot-based farmer support that translates AI diagnostics into actionable farming decisions, promoting accessible and farmer-friendly AI adoption [85].

To further enhance interactivity and modularity in plant phenotyping, Chen et al. (2024) introduced PhenoGPT. This prototype system integrates LLMs with task-specific computer vision models to support natural language-based interaction in plant phenotyping workflows. Built on GPT-3.5 Turbo, the system interprets user-provided text prompts, such as requests for disease classification or leaf segmentation, and automatically activates the appropriate vision model. The framework includes a Vision Transformer fine-tuned for cassava (*Manihot esculenta*) disease diagnosis, as well as a Mask2Former model for leaf instance segmentation. Benchmark evaluations showed that PhenoGPT accurately executed both tasks, whereas GPT-4o alone produced inconsistent results, highlighting the necessity of pretrained vision models for reliable trait extraction. This modular, user-friendly design demonstrates the potential of LLM-computer vision integration for accessible and scalable automated phenotyping [86].

Building on the goal of interactive vision-language pipelines, Madhavi et al. (2025) proposed a practical framework for automated plant disease phenotyping, which combines an image segmentation model (DeepLabV3+) with a GPT to detect and describe leaf diseases. Using a dataset of red raspberry (*Rubus idaeus*) leaves with annotated disease labels, the system identifies affected regions and generates textual descriptions of the observed symptoms. The segmentation model highlights diseased areas on the leaf surface using pixel-level classification, where small clusters of affected pixels are mapped amid essentially healthy tissue, typical of early-stage or localized plant diseases. These segmented regions are then used to guide GPT in producing descriptions that identify likely disease causes, such as rust, blight, or powdery mildew. Although the initial outputs resembled incomplete prompts, refinement of the input format is expected to improve diagnostic detail and accuracy. Each processing step was completed in approximately 2 s, suggesting potential for real-time agricultural applications. By combining visual symptom recognition with accessible textual output, this approach offers a promising tool for high-throughput, scalable, and user-friendly leaf-level plant stress phenotyping, especially in low-expertise settings [87].

Advancing multimodal integration, Zhu et al. (2025) developed a robust AI framework for potato disease management, combining MSC-TextCNN, CT-CNN, and MSC-ResViT models to analyze visual, textual, and statistical features. This ensemble achieved a classification accuracy of 98.43 % on the Potato Leaf Disease Dataset (Muhammad, 2021), which includes five categories: healthy, general early blight, severe early blight, general late blight, and severe late blight. The framework outperformed single-modal models and was successfully generalized to other *Solanaceae* crops, achieving 99.20 % accuracy on the tomato dataset and 97.30 % on the eggplant dataset. To operationalize the system in real-world settings, the authors introduced PotatoGPT, an online platform that integrates the detection results with GPT-4 to generate disease-specific control strategies. This study highlights the potential of vision-language integration to enable scalable, real-time, and field-ready plant disease diagnosis and decision support [88].

Cross-study comparison reveals that image-based plant stress diagnostics have rapidly advanced through the integration of lightweight CNNs, transformer-based VLMs, and LLMs, converging on key capabilities in multimodal reasoning, real-time inference, and field-level deployment. General object detection models, such as YOLOPC and MobileNetV2, demonstrate high accuracy (94.5–99 %) with reduced computational cost, enabling mobile and embedded applications. In contrast, transformer-based systems and multimodal pretraining frameworks consistently achieve ≥94 % accuracy by aligning textual and visual features through contrastive learning or self-attention. Benchmarks such as AgEval provide standardized datasets across 12 tasks, validating that few-shot models (e.g., GPT-4o) outperform in plant lesion and stress classification (F1: ∼74 %), whereas zero-shot models remain limited (F1: ∼50 %) without the use of curated prompts. Multimodal systems integrating LLMs with computer vision models (e.g., GPT + DeepLabV3+ and Mask2Former) support real-time segmentation and description of leaf-level symptoms. Ensemble and hybrid approaches further enhance classification accuracy (up to 98.43 %) and generalizability across crops. However, limitations persist, including model hallucination, dependency on dataset quality, and the need for domain-specific prompt engineering or fine-tuning of vision models. Collectively, these systems underscore the growing maturity of interactive, multimodal VLMs for plant stress identification, with future efforts needed in dataset expansion, robust field validation, and user-centered optimization for scalable agricultural deployment.

### 4.2 Text mining

LLMs have demonstrated significant potential in precision agriculture, particularly in crop monitoring and disease detection. By leveraging their ability to process and analyze vast amounts of textual data, LLMs can extract valuable insights from farmers’ reports and scientific literature, enabling the identification of patterns associated with crop diseases [89]. Integrating LLMs with other technologies further enhances their applicability in the agricultural sector.

Slimani et al. (2023) reviewed the integration of AI and drone technology in agriculture, primarily focusing on image-based disease detection using CNNs. While their study centered on visual analysis, it highlights the potential of combining LLMs with image-based approaches. LLMs can process and interpret textual descriptions of plant symptoms captured by drones, complementing visual data to enhance disease detection accuracy [90].

Zhang et al. (2024) investigated the application of LLMs in spatial trajectory pattern mining, illustrating how GPT-4 and Claude-2 can identify anomalous mobility behaviors and generate plausible explanations for their assessments. This capability could be adapted to track the spread of plant pathogens across agricultural regions, facilitating early intervention and targeted preventive measures [91].

In the context of phenotype recognition, Yang et al. (2024) developed PhenoBCBERT and PhenoGPT, two LLM-based models designed to expand the vocabulary of Human Phenotype Ontology terms. While their work focused on genetic diseases, the methodology could be adapted for crop phenotypes and disease symptoms [92]. These models exhibited superior performance in identifying a broader range of phenotype concepts compared to traditional tools, suggesting that LLM-based approaches could enhance the detection and characterization of crop diseases from textual descriptions.

Zhao et al. (2023) introduced ChatAgri, a framework leveraging ChatGPT models for agricultural text classification, addressing key challenges such as limited annotated training data, poor cross-linguistic domain transferability, and the high computational costs associated with fine-tuned models. Given the vast and continuously growing body of agricultural news, reports, and research, AI-driven text classification is crucial for efficiently organizing and accessing agricultural knowledge. Their study systematically explored ChatGPT's capabilities in agricultural informatization, evaluating various prompting strategies, answer parsing techniques, and model variants (e.g., GPT-3.5 and GPT-4). The findings demonstrated that ChatGPT achieves competitive performance without requiring additional training on domain-specific datasets. Notably, ChatGPT outperformed fine-tuned Pre-Trained Language Models (PLMs) in few-shot scenarios and exhibited strong cross-linguistic transferability, making it a viable solution for multilingual agricultural knowledge management. Beyond text classification, Zhao et al. (2023) underscored the broader implications of ChatGPT-driven AI systems in agricultural applications, including their role in enhancing digital transformation in precision agriculture, improving crop management, and optimizing production quality. By establishing ChatAgri as a scalable AI paradigm, their study lays the foundation for next-generation AI applications in agricultural information systems, contributing to the advancement of AI-powered sustainable agriculture [34].

Advancing multimodal integration, Zhao et al. (2024) developed an advanced system for Russian olive (*Elaeagnus angustifolia*) disease detection, fusing LLMs, Graph Neural Networks (GNNs), agricultural KGs, and Neural-Symbolic (NeSy) reasoning. Their model achieved 94 % precision, 92 % recall, and 93 % accuracy, significantly outperforming traditional methods. Through graph-based reasoning and dynamic attention, this system integrates structured and unstructured data to improve plant disease diagnosis. It combines a KG Encoder for structured knowledge, an LLM Encoder for textual data, and a Joint Reasoning Layer fusing both for decision-making. This approach enhances diagnostic accuracy and supports intelligent agricultural management [89].

Expanding the role of domain-adapted NLP in agricultural research, Khey et al. (2025) developed PlantDeBERTa, a domain-specific language model designed to extract structured information from plant stress literature. Built on the DeBERTa architecture, the model was fine-tuned using a manually annotated corpus of 142 abstracts focused on lentil (*Lens culinaris*) stress responses, capturing key biological entities such as plant species, abiotic and biotic stresses, and response types (e.g., molecular, physiological, agronomic). Following Crop Ontology standards and BIO tagging, the model achieved superior performance in NER, with a macro F1-score of 82.69 %, outperforming general and biomedical models such as BERT, BioBERT, and DistilBERT. These results highlight the importance of domain-specific pretraining for accurately identifying stress-related traits in the crop science literature. PlantDeBERTa sets a new benchmark for applying NLP tools in automated literature mining and trait annotation within plant phenotyping research [93].

Finally, Dofitas et al. (2025) proposed a weighted voting ensemble integrating Agricultural-BERT, Meta-LLaMA 3.1, and BERT-based uncased models to enhance agricultural knowledge retrieval. Leveraging domain-specific data and weighted voting, the ensemble model improves query performance and generates more accurate, context-aware outputs than individual models. Leveraging domain-specific data and weighted voting, the ensemble model achieved 93 % accuracy, along with high BLEU (53.8 %) and ROUGE (70 %, 55 %, and 61 %) scores, significantly improving query response quality and contextual relevance over individual models. This offers a robust LLM-based decision-support tool for agricultural management [94].

Cross-study comparison reveals that LLM-based agricultural text mining leverages three core strengths: domain adaptation, multimodal integration, and few-shot generalization. General-purpose models such as ChatAgri offer strong few-shot performance and cross-linguistic flexibility without fine-tuning, while domain-specific models such as PlantDeBERTa, fine-tuned on annotated abstracts, achieve high entity recognition accuracy (macro F1-score: ∼83 %) using ontology-aligned tagging. Ensemble approaches combining Agricultural-BERT, Meta-LLaMA 3.1, and BERT achieve a retrieval accuracy of 93 %, while multimodal systems integrating LLMs with KGs and GNNs reach similar accuracy through the fusion of structured and unstructured data. Additional applications include expanding phenotype ontologies and mining spatial trajectories using generative models. Despite these gains, key challenges persist: limited biological specificity in general models, high annotation demands for domain-specific ones, and opaque decision-making. These trends indicate the emergence of scalable, interpretable LLM frameworks with significant potential for robust, real-world agricultural applications.

### 4.3 Decision support systems

The integration of VLMs and LLMs into agricultural systems advances decision support frameworks by moving beyond perception-level inference toward high-level, context-aware recommendations for managing plant stress. While image identification and classification (Section 4.1) and text mining (Section 4.2) involve lower-level decisions focused on recognizing patterns or extracting knowledge, high-level decision support encompasses the synthesis of multimodal evidence into tailored agricultural advice, management strategies, and dynamic responses informed by environmental and field data. By leveraging advanced NLP capabilities, LLMs such as GPT-4 and BERT offer robust tools for analyzing large volumes of unstructured agricultural data, including research reports, field records, and expert guidelines [95,96]. These models can automatically extract critical information on disease characteristics, influencing factors, and management methods, thereby enabling evidence-based interventions that directly inform crop protection and plant stress mitigation strategies.

In particular, LLMs show significant promise in disease prevention and pest management, where they can analyze complex disease transmission pathways and recommend context-specific control strategies [39]. For example, LLMs can generate tailored pesticide recommendations and intervention measures based on real-time environmental and disease data, helping farmers reduce crop losses and optimize yield and quality [71]. The utility of LLMs extends further into agricultural advisory systems, where intelligent question-answering platforms powered by LLMs provide dynamic, real-time knowledge services to farmers and practitioners [72,73]. Such systems enable users to pose natural language questions about disease symptoms, treatment options, or preventive measures, with LLMs providing contextually relevant and scientifically grounded responses. Additionally, these platforms can offer personalized agronomic advice by processing farm-specific information, thereby enhancing decision-making and operational efficiency [97].

Recent studies have also evaluated the performance of LLMs in generating accurate and actionable pest management recommendations. Yang et al. (2024) examined the feasibility of LLMs such as GPT-4 for pest control decision support, focusing on the quality and reliability of AI-generated advice. Given the context-dependent nature of pest management, the study introduced an innovative evaluation framework employing GPT-4 as an automated scoring system to assess AI-generated recommendations across coherence, fluency, logical consistency, comprehensibility, relevance, and exhaustiveness. Moreover, an expert system based on scientifically validated crop threshold data was used as a factual baseline to benchmark the AI-generated outputs. The results demonstrated that GPT-4 consistently outperformed Google's FLAN and GPT-3.5 models across most evaluation metrics, particularly excelling in logical reasoning and comprehensibility. Furthermore, instruction-based prompting combined with domain-specific agricultural knowledge significantly enhanced the response accuracy of the model, reaching 72 % accuracy in pest management scenarios [71].

Despite these notable advancements, several critical limitations continue to hinder broader application. LLMs often produce overly generic suggestions and show reduced sensitivity to negative or edge cases, highlighting the need for continued model refinement and targeted fine-tuning to ensure specificity and practical relevance [71]. Future research should focus on optimizing prompting strategies to improve the contextual accuracy of LLM-generated pest and disease management advice. This includes the integration of detailed, pest-stage-specific dosage guidelines, treatment intervals, and localized monitoring protocols to ensure actionable and precise recommendations.

Moreover, aligning LLM-generated outputs with expert-validated knowledge and real-time environmental data is essential for enhancing reliability and applicability in diverse agricultural contexts. As summarized in Table 1, which overviews key LLM applications in plant stress identification and decision-making, integrating continuous expert feedback and adaptive learning mechanisms will be vital for deploying LLM-driven tools that meet the practical needs of farmers. Collectively, these advances underscore the potential of LLMs and VLMs to serve as adaptive, intelligent decision-support systems, streamlining agricultural management processes and contributing to the broader goals of sustainable and resilient precision farming.

**Table 1:** Applications of VLMs in plant stress identification and management.

Phenotyping Task	Model/Architecture Used	Target Stress Application	Performance Metrics	Key Innovations/Features	Reference
**Image Identification and Classification**	ITLMLP	Cucumber (*Cucumis sativus*) leaf diseases (anthracnose, powdery mildew, downy mildew, target spot)	Accuracy: 94.84 %	Combines image-text contrastive learning, image self-supervised learning, and label-guided distance measurement in a shared feature space	[80]
	GPT-4 with YOLOPC	Detection of agricultural pests and diseases, diagnostic report generation	YOLOPC: 94.5 % accuracy, AP 80.1 %; GPT-4: 90 % accuracy	Lightweight model (0.75M parameters, 1.4 GFLOPS), outperforms YOLOv5-nano, integrates vision and text for holistic diagnostics	[53]
	MobileNetV2 with LLMs	Early crop disease detection and tailored recommendations	Accuracy: 97 %–99 %	Mobile app for rural deployment integrates LLMs for prevention and treatment advice, emphasizes scalability, and XAI	[81]
	Gemini-pro-1.5, Claude-3.5-sonnet, and GPT-4o	Multimodal benchmark for plant stress phenotyping (identification, classification, quantification)	Zero-shot (F1: 50.45 %–82.98 %); Few-shot (F1: 73.37 %–95.94 %); NMAE: 12.78 %	AgEval benchmark excels in diverse tasks, highlights example relevance, and model stability	[82]
	Transformer-based VLM	Multimodal plant disease classification and diagnostics	Precision: 95 %, Recall: 92 %, Accuracy: 94 %	Outperforms traditional models (e.g., AlexNet, ResNet), leverages self-attention for cross-modal optimization	[83]
	GPT-4-based multimodal system	Plant disease diagnosis in integrated pest management (IPM)	Not specified	Integrates image recognition with NLP, uses the Korean plant/pesticide registry, and enhances early detection	[84]
	YOLOv8 with RAG and LLMs	Coffee (*C. arabica*) leaf disease detection and context-aware diagnosis	Not specified	Addresses LLM limitations (e.g., hallucinations) with RAG, supports real-time monitoring and scalability	[39]
	Langchain Llama with GANs and CNNs	Crop disease recognition and farmer-friendly recommendations	Not specified	Uses GANs for data augmentation, CNNs for classification, and a chatbot interface for accessibility	[85]
	PhenoGPT (LLM + Vision Transformer + Mask2Former)	Cassava (*M. esculenta*) disease diagnosis, leaf segmentation	Not specified	Integrates LLMs with task-specific computer vision models for natural language-based interaction in phenotyping; GPT-3.5 Turbo interprets text prompts and activates vision models for specific tasks	[86]
	DeepLabV3+ (image segmentation) + GPT (language generation)	Red raspberry (*R. idaeus*) leaf disease	Not specified	Segments diseased areas and generates textual descriptions of symptoms; detects early-stage localized plant diseases for high-throughput phenotyping	[87]
	MSC-ResViT, MSC-TextCNN, CT-CNN, and GPT-4	Potato (*S. tuberosum*) disease detection and management	MSC-ResViT: 92.15 % accuracy; MSC-TextCNN: 96.86 % accuracy; CT-CNN: 74.87 % accuracy; Multimodal framework: 98.43 % overall accuracy	Multimodal AI framework combining visual features, textual descriptions, and image-derived statistical features; online diagnosis platform (PotatoGPT)	[88]
**Text Mining**	LLMs (implied) with CNNs	Complementing drone-based image analysis with textual symptom interpretation	Not specified	Focus on image-based detection suggests LLM potential for textual data integration	[90]
	GPT-4 and Claude-2	Spatial tracking of plant pathogen spread	Not specified	Adapting spatial trajectory mining for agriculture enables early intervention	[91]
	PhenoBCBERT and PhenoGPT	Phenotype recognition adaptable to crop disease symptoms	Superior to traditional tools	Expands phenotype vocabularies, potential for crop disease characterization	[92]
	ChatGPT (GPT-3.5, and GPT-4)	Agricultural text classification for disease knowledge management	Outperforms fine-tuned PLMs in few-shot scenarios	ChatAgri framework, strong cross-linguistic transferability, scalable for agricultural informatization	[34]
	LLMs, Agricultural KGs, GNNs, and neural-symbolic reasoning	Russian olive (*E. angustifolia*) disease detection	Precision: 94 %, Recall: 92 %, Accuracy: 93 %	KG Encoder with GNNs and MLPs, LLM Encoder with Transformer-based models, Dynamic Attention Mechanism, Joint Reasoning Layer	[89]
	PlantDeBERTa (DeBERTa architecture)	Lentil (*L. culinaris*) stress responses	Macro F1-score: 82.69 %	Domain-specific model fine-tuned on a manually annotated corpus; superior performance in NER; outperforms BERT, BioBERT, and DistilBERT	[93]
	Agricultural-BERT, Meta-LLaMA 3.1, and BERT-based uncased models	Agricultural knowledge retrieval	93 % accuracy, BLEU: 53.8 %, ROUGE-1: 70 %, ROUGE-2: 55 %, ROUGE-L: 61 %	Weighted voting ensemble method, domain-specific data integration, optimized query performance, and response generation	[94]
**Decision Support Systems**	GPT-4	Pest management advice, evaluation, and generation	72 % accuracy in pest management advice	Automated scoring system, integrates expert thresholds, excels in reasoning, but needs specificity refinement	[71]

## 5 Challenges and limitations

While the integration of LLMs and vision-based AI in agriculture presents transformative potential, several challenges and limitations must be addressed to ensure effective plant stress management. These challenges span technical, implementation, and ethical dimensions, affecting the scalability and reliability of multimodal AI systems in agricultural applications.

### 5.1 Technical challenges

Despite the remarkable advances of large multimodal VLMs and LLMs, their application in plant stress management remains constrained by several persistent technical challenges. A primary obstacle is the limited availability of high-quality, domain-specific agricultural datasets, which significantly restricts model accuracy and generalizability. Unlike general-purpose datasets, agricultural data covering complex phenomena such as plant disease symptoms, pest dynamics, and environmental stressors are often fragmented, unstructured, or entirely absent, especially for rare crops and localized stress factors [34]. This scarcity is particularly acute for emerging pests and diseases, where representative datasets may be completely unavailable.

The lack of sufficient data has several downstream consequences. Many existing agricultural datasets rely on silver-standard annotations or limited-source inputs, which introduce biases and reduce robustness [98]. Data fragmentation and inconsistency, arising from variability in environmental conditions, farming practices, and crop species, further impede model training and deployment. As a result, large models that depend on extensive, high-quality corpora for pre-training and fine-tuning cannot be fully optimized for agricultural applications. This restricts their ability to generalize across environments and to perform quantitative, context-specific reasoning beyond generic text-based outputs [2]. For instance, a VLM trained on limited greenhouse imagery may perform well in controlled settings but fail to recognize diverse field environments, while an LLM trained on sparse agricultural corpora may misinterpret phenotypic traits or regional practices in underrepresented regions.

Exacerbating this issue is the limited pre-training of LLMs on agricultural datasets. While multimodal AI models have demonstrated superior generalization compared with traditional expert systems, they remain vulnerable to biases, error propagation, and imbalances inherent in text- and image-based sources [99]. Moreover, the dynamic and evolving nature of agricultural terminologies, spanning regional dialects, synonyms, and context-dependent jargon, is poorly captured by models predominantly trained on English-language corpora [34]. This linguistic limitation further undermines the applicability of LLMs in diverse agricultural contexts, particularly in data-scarce and underrepresented regions.

To address these data-related challenges, several strategies are being pursued to mitigate data scarcity and improve dataset quality, completeness, and interoperability. Standardization initiatives aim to establish consistent data collection protocols and formatting conventions, facilitating the integration of heterogeneous sources such as weather logs, sensor data, and satellite imagery [76,100]. In parallel, data enrichment techniques, including spatial-temporal interpolation, multi-source data fusion, and the incorporation of historical or global datasets, help mitigate gaps and enhance representativeness. Synthetic data generation has also emerged as a promising approach to expand limited agricultural datasets. For instance, conditional generative adversarial networks (CGANs) can generate synthetic images from preprocessed agricultural inputs, effectively addressing problems of class imbalance, noisy labels, and data scarcity to improve model prediction accuracy. Abbas et al. (2021) demonstrated this by producing synthetic tomato leaf images with CGANs, which enhanced classification performance compared with training on raw datasets alone [101]. Validation and cleaning processes, which involve both automated pipelines and expert-led manual reviews, are crucial for ensuring dataset accuracy and usability. Additionally, crowdsourcing approaches and local expert collaboration contribute real-time, context-specific knowledge that refines the relevance and precision of agricultural datasets [79]. Collectively, these initiatives play a pivotal role in overcoming the fragmentation and unreliability of current agricultural data, thereby enhancing the effectiveness of LLM and VLM applications in precision agriculture.

Computational costs present another formidable challenge. The training, fine-tuning, and deployment of LLMs such as ChatGPT, LLaMA, and DeepSeek demand substantial computational resources and technical expertise, restricting their accessibility for researchers, extension workers, and farmers, particularly in low-resource settings [102]. Real-time agricultural applications, such as in-field disease diagnostics and precision irrigation, require efficient, low-latency processing, often incompatible with the high infrastructure demands of current LLMs [74]. Additionally, the large-scale energy consumption associated with these models raises sustainability concerns, highlighting the need for energy-efficient architectures and optimization techniques.

Another critical limitation lies in image recognition and interpretation, which are fundamental to LLM-assisted plant stress diagnosis. Misclassifications in agricultural imaging, stemming from insufficient training data or visual variability, can lead to erroneous diagnoses and ineffective interventions. The integration of heterogeneous agricultural data sources, including satellite imagery, Internet of Things (IoT) sensors, weather data, and farm logs, exacerbates this challenge due to incompatible data formats and standards, ultimately diminishing the accuracy and actionability of LLM-generated insights [76].

Beyond data and computational challenges, model reliability remains an unresolved issue. LLMs are prone to hallucinations, generating fabricated or misleading content that appears plausible [103]. These hallucinations often stem from biased training data or limited diversity in verified agricultural sources. Techniques such as RAG offer a promising solution, combining LLM outputs with real-time, external knowledge bases to enhance factual accuracy [44,104]. Moreover, user-side interventions, such as refining prompt structures and adjusting generation parameters such as temperature, can partially mitigate unreliable outputs [105,106].

Another underappreciated challenge is catastrophic forgetting, where models lose previously learned knowledge when adapting to new information [107]. This is particularly problematic in agriculture, where models must account for both historical and emerging stress factors. Advanced techniques such as Elastic Weight Consolidation (EWC), inspired by biological memory mechanisms, offer a structured approach to preserving essential knowledge during continual learning [108,109].

Finally, although recent advances in automatic prompt generation and instruction tuning have improved the domain specificity of GPT-based models for agriculture, these methods require careful calibration to avoid inconsistencies [99]. Current models also struggle with agricultural ontologies and hierarchical taxonomies, limiting their capacity to interpret complex relationships among plant species, diseases, and environmental variables [110].

### 5.2 Implementation challenges

Beyond technical limitations, the integration of LLMs/VLMs and AI-driven tools into practical agricultural systems presents significant implementation challenges that span economic, infrastructural, and institutional domains. A critical barrier to widespread adoption is the high cost and complexity of AI technologies, which often require advanced computational infrastructure that is unavailable in rural and resource-limited settings [71,111]. Although high-capacity models such as GPT-4 and LLaMA demonstrate impressive performance in multimodal reasoning tasks, their dependence on cloud-based GPUs and substantial memory makes them more suitable for centralized precision agriculture platforms with stable internet access and cloud support [102]. Conversely, lightweight architectures such as YOLOPC, or ITLMLP offer reduced computational overhead and faster inference speeds, making them more appropriate for edge-based deployment in real-time, offline, or low-resource farm environments [53,80]. Thus, model selection should align with the target implementation context, balancing accuracy with cost, infrastructure, and latency requirements to ensure practical scalability and usability for farmers and agronomists.

The implementation of VLMs in agriculture introduces additional domain-specific challenges. Pretrained VLMs are typically trained on general-purpose web data, which limits their capacity to identify fine-grained plant stress features or subtle phenotypic variations [112,113]. Moreover, the lack of high-quality, labeled agricultural image-text datasets hampers the fine-tuning and evaluation of these models. Agricultural expertise is also specialized and scarce, complicating the curation of benchmark datasets and validation workflows [[113], [114], [115]]. Finally, proprietary VLMs pose access and customization barriers that reduce transparency and limit domain adaptation (X. Yang et al., 2024), especially in low-resource settings [116].

Data heterogeneity and complexity present additional obstacles. Agricultural data is inherently multifaceted, encompassing sensor-generated measurements, textual farmer reports, satellite imagery, soil and weather records, and other relevant agronomic data. Current LLMs struggle to process and integrate these diverse, context-rich datasets, thereby limiting their ability to generate accurate and actionable insights [76,100]. Moreover, the fragmented and decentralized nature of agricultural data collection, conducted independently by farmers, agronomists, researchers, and governmental bodies, results in datasets that are inconsistent in format and quality, complicating their aggregation and use in AI systems [117]. The absence of standardized data collection and sharing protocols impedes effective AI integration and weakens platform interoperability.

Further exacerbating these challenges is the limited availability of high-quality, comprehensive agricultural datasets necessary for training LLMs. Existing datasets are often incomplete, inconsistent, or inaccessible, with significant gaps in region- and crop-specific information [76,100]. Given the geographical variability in agricultural contexts, such as differences in climate, soil composition, pest pressure, and farming practices, AI models trained on narrow datasets struggle to generalize across diverse farming environments [118]. Without standardized global datasets, LLMs cannot deliver the level of localized precision required for practical decision-making in plant stress management.

Infrastructure limitations also pose a formidable barrier to AI adoption, particularly in rural areas with inadequate power supplies, wireless connectivity, and computing resources [119]. Even when data is available, latency and bandwidth issues hinder real-time analytics and responsiveness, capabilities crucial for in-field applications such as pest outbreak prediction or precision irrigation. The integration of AI and IoT technologies introduces further complications related to data flow management, security, and scalability, which necessitate tailored solutions such as edge computing for local data processing and adaptive AI models optimized for low-resource settings [120].

Another pivotal factor influencing AI adoption in agriculture is institutional and governmental support. Studies have shown that financial incentives, policy frameworks, and educational initiatives significantly influence the willingness of farmers to adopt AI-driven tools [121,122]. Furthermore, the familiarity of farmers with digital technologies and their alignment with local agricultural practices are essential for effective AI implementation. Without context-specific training and capacity-building efforts, even well-designed AI tools may face resistance or improper use [123].

Notably, the scalability and reliability of LLMs in dynamic agricultural environments remain unresolved. LLM-based tools, such as ChatGPT-derived models for crop disease diagnosis, often perform well in controlled research settings yet fail to maintain contextual relevance and accuracy in real-world applications, particularly when confronted with unfamiliar crops, regional terminologies, or emerging stressors [124]. This issue is compounded by the static nature of most LLMs, which are trained on existing datasets and lack mechanisms for adaptive learning in response to novel plant diseases, evolving pest threats, or shifting climatic conditions [125].

Addressing these multifaceted implementation barriers requires a comprehensive and collaborative approach. Enhancing data collection infrastructure through the widespread deployment of IoT devices, remote sensing technologies, and standardized data-sharing protocols can ensure more consistent and representative datasets [126]. Furthermore, fostering collaboration among farmers, researchers, policymakers, and technology providers, including crowdsourcing initiatives and community-based data contributions, is crucial for enhancing data diversity, quality, and accessibility. Such collaborative frameworks enhance the comprehensiveness of datasets and promote the trust and usability of AI solutions among end-users.

Ultimately, overcoming these implementation challenges will significantly improve the scalability, reliability, and practical applicability of LLM-based and AI-driven systems for plant stress management, paving the way for more resilient and data-driven agricultural practices worldwide.

### 5.3 Ethical considerations

The integration of LLMs and multimodal AI systems in agriculture introduces a complex set of ethical considerations, spanning data privacy and ownership issues, algorithmic fairness, misinformation risks, workforce displacement, and trust in LLM-generated recommendations. Effectively addressing these ethical challenges is essential to promoting the responsible and equitable implementation of LLM-driven solutions in plant stress management.

A central concern is data privacy and ownership, particularly when LLMs are fine-tuned on sensitive agricultural datasets, including farm-level production data, pest and disease outbreaks, and detailed soil analyses. Farmers frequently express concerns about unauthorized access, data misuse, and potential exploitation by third parties, which reduces their willingness to share crucial data [127]. Ensuring that farmers and agricultural organizations retain control over their data is crucial to fostering trust and encouraging participation in LLM-driven ecosystems. Emerging frameworks, such as privacy-preserving synthetic data generation methods, offer promising pathways to safeguard sensitive information while enabling model development [128]. Nonetheless, continuous refinement of data protection protocols is essential to keep pace with evolving cybersecurity threats [129].

Algorithmic fairness and bias present another profound ethical challenge. LLMs trained on imbalanced or incomplete datasets risk producing outputs that disproportionately favor well-represented crops, regions, and languages while underperforming for marginalized communities, underrepresented crops, and diverse farming practices [34]. This raises concerns about exacerbating existing inequalities in agricultural access and benefits, particularly for smallholder farmers in developing regions. Even when explicit biases are absent in training data, research has shown that subtle and systemic biases can still emerge in model outputs [[130], [131], [132]]. Addressing these risks requires carefully curated, diverse datasets and bias-mitigation strategies throughout the model development and deployment process.

The risk of misinformation generated by AI systems is another critical ethical issue. AI models, including LLMs, are prone to hallucinations, generating factually incorrect outputs yet presented with unwarranted confidence [103]. In agriculture, erroneous recommendations, such as inappropriate pest management strategies or incorrect diagnoses of plant diseases, can lead to severe consequences, including crop losses, environmental damage, and food insecurity. The problematic transition to organic farming in Sri Lanka, reportedly influenced by flawed policy recommendations, exemplifies the potential societal risks of misinformed AI guidance [133]. Thus, robust validation frameworks, continuous model evaluation, and real-time error monitoring are crucial in preventing misinformation [134].

Workforce displacement due to AI automation in agriculture raises further ethical and socioeconomic concerns. AI-driven tools capable of automating crop monitoring, disease detection, and precision farming may reduce the need for traditional manual labor, potentially impacting rural employment [55,135,136]. While AI can enhance efficiency, strategic interventions, such as retraining and upskilling programs, are necessary to ensure that the agricultural workforce transitions smoothly into AI-integrated roles, thereby preventing the widening of economic disparities [55,137,138].

Ultimately, the trust and interpretability of AI systems are crucial for their widespread adoption and responsible use. Many LLMs operate as opaque “black boxes,” making it difficult for users to understand how conclusions are reached [139]. This lack of transparency erodes the confidence of stakeholders, particularly when AI recommendations influence high-stakes decisions that impact food security, environmental sustainability, and economic viability [140]. The variability in datasets, model parameters, and computational environments also complicates reproducibility and validation. To address these issues, research in XAI focuses on developing interpretable models that clarify decision-making processes [141]. Techniques such as saliency maps, which visually highlight key features influencing AI decisions, and adversarial testing, which assesses AI resilience to data perturbations, offer promising pathways for improving model transparency and trustworthiness [142,143].

Effectively navigating these ethical considerations demands a multifaceted strategy. This includes advancing model transparency and interpretability, improving dataset diversity and quality, enforcing rigorous data privacy standards, and establishing clear regulatory frameworks. Equally important is collaborative engagement among stakeholders, including farmers, agronomists, policymakers, AI developers, and ethicists, to ensure that AI-driven agricultural innovations align with broader sustainability goals, equity, and food security. Through such comprehensive efforts, the responsible and ethical deployment of AI in agriculture can be realized, unlocking its full potential to address global challenges in plant stress management.

## 6 Future directions

**Fig. 2:**
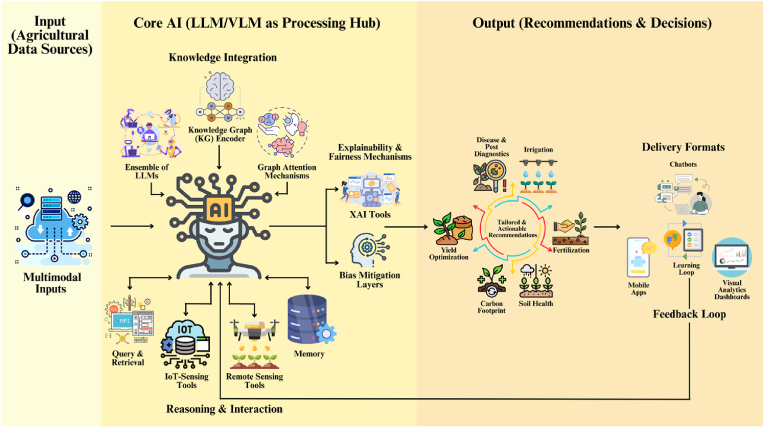
Proposed AI-driven framework for plant stress management leveraging LLMs and VLMs. The system integrates multimodal agricultural data sources (e.g., IoT and remote sensing tools) into a centralized AI hub combining ensemble LLMs, KGs, and graph attention mechanisms for domain-specific reasoning. Explainability and bias mitigation layers ensure transparency and fairness in AI-generated outputs. The framework supports real-time, context-aware decision-making for plant stress diagnostics, resource allocation (e.g., irrigation, fertilization), and sustainability monitoring (e.g., carbon footprint, soil health). Outputs are delivered via chatbots, mobile apps, and analytics dashboards, with a feedback loop for continuous system learning and adaptation. This architecture addresses future directions for scalable, interpretable, and inclusive AI-based precision agriculture.

The integration of multimodal LLMs with advanced image recognition and sensing technologies presents promising opportunities for improving plant stress management and broader agricultural applications. As AI-driven agriculture continues to evolve, key developments will focus on enhancing data collection, predictive analytics, and real-time decision support. As outlined in Fig. 2, a proposed AI-driven framework integrates an ensemble of LLMs, VLMs, KGs, and multimodal sensor data to enable real-time, context-aware diagnostics, resource optimization, and sustainable crop management. Future advancements will enable more precise crop monitoring, optimized resource management, and enhanced pest and disease detection, ultimately leading to more efficient and sustainable farming practices.

### 6.1 Advancements in ensemble and knowledge-based models

A promising future direction for enhancing AI-driven plant stress management lies in the development of ensemble learning techniques and knowledge-integrated frameworks, which address the limitations of single-model approaches in complex and variable agricultural environments.

Ensemble learning, which combines multiple LLMs with complementary strengths, improves accuracy, robustness, and adaptability in diagnosing and managing plant stress conditions [125,144]. Given the heterogeneous and high-variance nature of agricultural stress factors, ranging from biotic threats such as pathogens and pests to abiotic stressors such as drought and soil salinity, individual models often struggle to generalize effectively. By integrating diverse LLMs, ensemble models can synthesize multi-perspective insights, facilitating more comprehensive and reliable decision-support systems. These systems can analyze various data sources, including crop images, textual farmer reports, and sensor-derived environmental data, to deliver well-rounded and actionable recommendations.

Evidence from other domains demonstrates the superior performance of ensemble methods over individual models, including applications in AI-generated content detection and forecasting competitions, where ensemble systems have achieved human-level accuracy [145]. When applied to agriculture, ensemble models are expected to mitigate biases inherent in individual LLMs, ensuring more balanced and equitable recommendations across diverse agroecological contexts and farming communities.

In parallel, the integration of agricultural KGs into LLM architectures represents a critical advancement in domain-specific reasoning. KGs encode structured, relational representations of complex agricultural knowledge, capturing interactions among crop species, diseases, environmental stressors, and management practices [146]. By coupling LLMs with KGs, AI systems gain enhanced contextual understanding, enabling more precise and context-aware plant stress diagnosis and intervention recommendations [147].

Graph attention mechanisms can be employed to dynamically identify and prioritize the most relevant entities and relationships within large and complex datasets, further optimizing these hybrid models. This selective focus is especially valuable for targeted disease detection and stress response analysis, where context sensitivity is essential [148]. Moreover, the application of graph-based loss functions during training can enforce domain-specific constraints, guiding AI models toward outputs aligned with agronomic principles and real-world agricultural conditions [149].

Recent advancements such as ITK-Net, which successfully integrates multimodal data (images and text) with KGs, have demonstrated exceptionally high performance in agricultural disease identification, achieving precision rates exceeding 99 % [6]. Building upon such designs, LLM-KG ensemble architectures provide a pathway toward robust, explainable, and highly accurate plant stress management systems that can deliver nuanced insights, addressing the practical demands of real-world agriculture.

Together, ensemble learning and KG integration represent transformative approaches for overcoming current limitations in LLM-driven agriculture. By fostering more reliable, context-aware, and interpretable LLM models, these innovations hold the potential to significantly advance precision agriculture, sustainable crop management, and resilient food systems amid increasing environmental and biological stresses.

### 6.2 AI and IoT integration for precision agriculture

The convergence of LLMs, VLMs, and IoT technologies is poised to revolutionize precision agriculture by enabling near–real-time, adaptive, and context-aware decision-making. By integrating AI models with edge computing and smart IoT sensors, future agricultural systems can perform on-site analysis of multimodal data, including hyperspectral imagery, soil moisture, pH, and nutrient levels, without relying on constant cloud connectivity. This is especially critical in remote or connectivity-limited regions, where drones, field robots, and handheld devices equipped with VLMs can autonomously detect and differentiate plant stress symptoms with high accuracy [[150], [151], [152]]. The fusion of visual, textual, and numerical inputs enhances the explainability and responsiveness of AI-driven decisions for pest surveillance, irrigation scheduling, and stress mitigation. Ultimately, this integration supports the development of scalable, low-latency, and sustainable precision agriculture systems that improve resource efficiency and resilience across diverse farming environments.

A key frontier in this evolution is the integration of LLMs with proximal sensing technologies, facilitating near–real-time, field-level insights through handheld sensors, soil probes, and IoT-based measurement devices. These systems will enable localized detection of soil health fluctuations, water stress, nutrient deficiencies, and pest infestations, optimizing resource allocation and reducing unnecessary agricultural inputs [33]. Future advancements will focus on improving sensor precision, enhancing AI inference models, and developing adaptive LLM systems capable of continuously learning dynamically from real-time feedback to refine their recommendations [153]. Combined with edge computing, these systems will process data directly on-site, minimizing latency and facilitating instant decision-making, even in resource-constrained or remote farming environments.

Complementing proximal sensing and remote sensing technologies, including satellite-based hyperspectral imaging, Unmanned Aerial Vehicle (UAV)-mounted sensors, and geospatial analytics, will drive large-scale agricultural transformations. Integrating LLMs with autonomous drone fleets equipped with AI vision models will enable near–real-time assessments of crop stress and the early detection of plant diseases, nutrient imbalances, and climate-induced stressors [154]. The future trajectory of LLM-enhanced remote sensing envisions multimodal fusion frameworks that integrate satellite imagery, in-situ sensor data, and weather forecasts, creating holistic AI-driven models for precision crop monitoring and climate-resilient agriculture [155].

Crucially, LLMs integrated within IoT ecosystems will enhance near–real-time agricultural decision support systems by offering natural language insights tailored to localized conditions, including soil types, crop varieties, and microclimatic variables [156]. These context-aware AI models will enable dynamic and personalized recommendations, empowering farmers to make informed decisions about pest control, fertilization, and irrigation. The incorporation of edge-based AI processing will ensure that data privacy and security are upheld by reducing reliance on centralized cloud servers, thus mitigating cybersecurity risks and preserving farmer data ownership. This distributed architecture enables autonomous operation, making AI-driven insights accessible even in areas with limited internet connectivity, thereby expanding the reach of precision agriculture innovations.

A central advantage of AI-IoT integration lies in its capacity to implement context-aware routing strategies, which dynamically select the most computationally efficient and accurate AI models based on data complexity and available processing resources. This hierarchical AI processing framework, spanning edge, cloud, and distributed IoT networks, will optimize near–real-time responsiveness while maintaining low energy and computational demands. Future developments will prioritize lightweight, energy-efficient AI models capable of operating on low-power edge devices, ensuring the scalability and affordability of AI-driven agricultural solutions across diverse farming contexts [157].

Moreover, predictive analytics powered by AI- and IoT-based systems will enable proactive plant stress management. By continuously analyzing environmental and biological signals, LLM-based systems will anticipate emerging threats, such as pest outbreaks or water shortages, allowing farmers to implement timely interventions and avoid yield losses. These capabilities will be central to building climate-resilient farming systems that can adjust to increasingly unpredictable weather patterns and environmental challenges.

Despite these promising prospects, critical challenges must be addressed to realize the full potential of AI-IoT integration in agriculture. Data reliability and interoperability remain pressing issues, as fragmented data sources and incompatible formats hinder seamless integration and data sharing. The development of standardized data-sharing frameworks and interoperable platforms will be crucial to ensuring that AI systems can effectively aggregate and analyze diverse data streams. Additionally, energy-conscious AI model design must prioritize computational efficiency and sustainability, reducing the environmental footprint of AI-driven sensing technologies.

In the future, the integration of LLMs with VLMs, next-generation IoT, and advanced sensing technologies will enable the development of intelligent, self-learning agricultural systems capable of near–real-time environmental monitoring, predictive decision support, and autonomous intervention. This synergy will drive transformative improvements in crop yield forecasting, plant stress detection, and operational efficiency by combining multimodal data sources, such as image-based diagnostics, sensor readings, and contextual text inputs, into a unified decision-making framework. Enhanced early detection of pests and diseases through these AI-driven systems will facilitate timely and targeted responses, strengthening IPM strategies and reducing crop loss. Moreover, LLM- and VLM-powered platforms will generate tailored, plant-level recommendations for stress mitigation and input optimization, empowering farmers to adapt practices dynamically based on localized conditions. As these technologies become increasingly embedded in sensor-rich environments, they will provide deeper insights into plant-environment interactions, facilitating the emergence of sustainable, resilient, and inclusive precision agriculture systems that can address the escalating challenges of climate change and global food security [153].

### 6.3 Enhancing accessibility, explainability, and sustainability

As LLMs and multimodal AI systems become increasingly integrated into agricultural decision-making, ensuring accessibility and explainability is crucial for their practical, ethical, and widespread adoption. Future research must prioritize the development of cross-domain, multimodal, and multilingual LLM frameworks, integrating diverse data streams such as satellite imagery, field sensor outputs, climate models, and textual agronomic knowledge to provide a holistic and context-aware understanding of plant stress phenomena [110]. Given that plant stress is shaped by multifactorial and interdependent conditions, such comprehensive data integration is essential for generating accurate and actionable AI recommendations.

Multilingual AI development represents a cornerstone of future AI accessibility. Since agriculture is practiced globally across diverse linguistic and cultural landscapes, LLMs must accurately process and convey domain-specific knowledge in multiple languages. While recent initiatives, such as CulturaX, which encompasses 6.3 trillion tokens across 167 languages, mark significant progress [158], substantial gaps remain, particularly for low-resource languages. Current LLMs perform unevenly, favoring high-resource languages with abundant training data while underperforming in low-resource agricultural contexts [159]. Addressing this imbalance requires advancements in multilingual instruction tuning, cross-lingual transfer learning, and domain-specific translation capabilities. Fine-tuned multilingual LLMs have already demonstrated promising results in domains such as precision farming and crisis response [160,161], indicating strong potential for broader agricultural applications. As multilingual LLMs mature, their integration into collaborative platforms will be key to translating these technical advances into practical, farmer-centered applications.

AI-powered collaborative platforms will be critical for democratizing access to AI innovations. These platforms, underpinned by LLMs, will facilitate knowledge-sharing and participatory decision-making among farmers, researchers, and policymakers. Acting as intelligent intermediaries, LLMs can translate complex scientific findings into practical, region-specific recommendations, enabling farmers to implement evidence-based solutions [162]. Moreover, integrating intelligent search engines and personalized recommendation systems within these platforms will enable users to access curated agronomic insights and region-specific crop management techniques, as well as connect with experts [163]. By democratizing access through collaborative platforms, AI can also be harnessed to address broader sustainability challenges in farming systems.

Importantly, sustainability-focused AI frameworks will guide environmentally responsible farming. Future AI models will optimize productivity and analyze key sustainability indicators, including water use efficiency, soil health, biodiversity, and carbon footprints. By benchmarking farm operations against global sustainability standards and generating tailored recommendations, AI will enable farmers to align agricultural practices with broader environmental goals [163]. Training LLMs on large-scale datasets of sustainable agriculture practices will facilitate the development of context-specific sustainability assessments and reporting tools, advancing both economic resilience and sustainable land management.

Future progress will depend on collaborative efforts among researchers, industry leaders, policymakers, and farming communities to co-create scalable, robust, and ethically grounded AI systems. Multimodal AI must evolve beyond theoretical frameworks to become practical, trustworthy, and inclusive tools that address the real-world complexities of agriculture. Ultimately, the fusion of AI-driven analytics, sustainable farming practices, and inclusive technologies holds the potential to transform agriculture into a more resilient, efficient, and equitable sector capable of meeting the challenges of global food security and environmental change.

## 7 Conclusion

The integration of large multimodal VLMs into plant stress phenotyping marks a transformative shift in plant science, enabling the fusion of biological complexity with real-time, data-driven insight. By combining advanced visual recognition with contextual reasoning, VLMs enable the early detection, precise monitoring, and targeted analysis of plant stress phenotypes, thereby moving beyond the limitations of traditional assessment methods.

The rapid evolution of AI-assisted platforms is redefining the scope of plant science by integrating high-resolution imaging, sensor data, molecular profiles, and environmental metadata. LLM-based agents such as ChatGPT, Claude, Mistral, DeepSeek, and Gemini are extending these capabilities across the research pipeline, from genome annotation and trait prioritization to stress prediction and phenotypic classification. These developments are not only accelerating discovery but also shifting the research frontier toward formulating new, creative questions that leverage the inferential power of AI.

As VLMs and LLMs continue to advance, their central role in precision agriculture, real-time phenotyping, and adaptive decision-making is becoming increasingly evident. However, to fully realize this potential, several domain-specific challenges must be addressed. Some challenges are likely to be alleviated as newer LLMs and VLMs benefit from larger training corpora, expanded context windows, and greater computational resources, for example, handling long-sequence multimodal inputs or achieving baseline improvements in reasoning and generalization. Other challenges, however, are unlikely to be solved by scale alone. These include the limited availability of large, diverse, and well-annotated agricultural datasets; the need for transparent interpretability and scientific validation; and ensuring global equity in AI access. Particularly, the widening technological divide between well-resourced and resource-limited regions calls for collaborative, open-access data ecosystems and sustainable AI deployment strategies.

Moreover, the integration of RAG with VLMs and LLMs elevates plant phenotyping from visual recognition to knowledge-grounded reasoning. By incorporating domain-specific retrieval mechanisms, RAG enhances diagnostic precision and adaptability to complex or novel stressors, such as visually overlapping symptoms like chlorosis and wilting, thereby improving overall accuracy. Multimodal systems that jointly analyze image data, sensor outputs, and metadata can simulate expert-level reasoning, offering fine-grained, context-aware stress differentiation in dynamic field environments.

To unlock the full potential of AI in plant phenotyping, coordinated efforts between plant biologists and AI researchers will be crucial in steering responsible innovation. Realizing the contribution of AI-driven phenotyping systems to global food security and climate resilience demands not only cutting-edge technological development but also inclusive governance frameworks and strategic foresight. Looking forward, the research outlook should balance optimism about rapid gains from scaling LLMs with a recognition that domain-specific bottlenecks, particularly data scarcity, interpretability, and equitable access, require targeted scientific and policy solutions.

As one of the first studies to consolidate the role of VLMs and LLMs in plant stress phenotyping, this review outlines a promising framework that integrates multimodal AI for high-throughput trait analysis, accessible diagnostics, and intelligent decision-making, laying the groundwork for more scalable and user-centric agricultural technologies.

## Author contributions

X.F. and E.M conceptualized the manuscript; E.M. and I.I.M. prepared the initial draft; E.M., I.I.M., J.G., and X.F. reviewed and revised the manuscript. All authors have read and agreed to the published version of the manuscript.

## Institutional review board statement

Not Applicable.

## Informed consent statement

Not Applicable.

## Data availability statement

Not Applicable.

## Funding

This research was supported by the Biological Breeding-National Science and Technology Major Project of China (2023ZD040360301) and the National Natural Science Foundation of China (U21A20215 and 32488102).

## Conflicts of interest

The authors declare no conflict of interest.

## References

[1] Araus J.L., Cairns J.E. (2014). Field high-throughput phenotyping: the new crop breeding frontier. Trends Plant Sci..

[2] Li J., Xu M., Xiang L., Chen D., Zhuang W., Yin X., Li Z. (2024).

[3] Li Y., Wu X., Zhang Y., Zhang Q. (2022). CRISPR/cas genome editing improves abiotic and biotic stress tolerance of crops. Front. Genome Ed.

[4] Shiade S.R.G., Zand-Silakhoor A., Fathi A., Rahimi R., Minkina T., Rajput V.D., Zulfiqar U., Chaudhary T. (2024). Plant metabolites and signaling pathways in response to biotic and abiotic stresses: exploring bio stimulant applications. Plant Stress.

[5] Fahlgren N., Gehan M.A., Baxter I. Lights (2015). Camera, action: high-throughput plant phenotyping is ready for a Close-Up. Curr. Opin. Plant Biol..

[6] Zhou J., Li J., Wang C., Wu H., Zhao C., Teng G. (2021). Crop disease identification and interpretation method based on multimodal deep learning. Comput. Electron. Agric..

[7] Thakur P.S., Khanna P., Sheorey T., Ojha A. (2022). Trends in vision-based machine learning techniques for plant disease identification: a systematic review. Expert Syst. Appl..

[8] Shin J., Chang Y.K., Heung B., Nguyen-Quang T., Price G.W., Al-Mallahi A. (2021). A deep learning approach for RGB image-based powdery mildew disease detection on strawberry leaves. Comput. Electron. Agric..

[9] Gao R., Wang R., Feng L., Li Q., Wu H. (2021). Dual-branch, efficient, channel attention-based crop disease identification. Comput. Electron. Agric..

[10] Ferentinos K.P. (2018). Deep learning models for plant disease detection and diagnosis. Comput. Electron. Agric..

[11] Jadon S., Jadon A. (2020). An overview of deep learning architectures in few-shot learning domain. arXiv Prepr. arXiv2008.06365.

[12] Argüeso D., Picon A., Irusta U., Medela A., San-Emeterio M.G., Bereciartua A., Alvarez-Gila A. (2020). Few-shot learning approach for plant disease classification using images taken in the field. Comput. Electron. Agric..

[13] OpenAI, Achiam J., Adler S., Agarwal S., Ahmad L., Akkaya I., Aleman F.L., Almeida D., Altenschmidt J., Altman S. (2024). GPT-4 Technical Repor.

[14] Touvron H., Lavril T., Izacard G., Martinet X., Lachaux M.-A., Lacroix T., Rozière B., Goyal N., Hambro E., Azhar F. (2023).

[15] Mathimani T., Mallick N. (2018). A comprehensive review on harvesting of microalgae for biodiesel - key challenges and future directions. Renew. Sustain. Energy Rev..

[16] Diwan T., Anirudh G., Tembhurne J.V. (2023). Object detection using YOLO: challenges, architectural successors, datasets and applications. Multimed. Tool. Appl..

[17] Dosovitskiy A., Beyer L., Kolesnikov A., Weissenborn D., Zhai X., Unterthiner T., Dehghani M., Minderer M., Heigold G., Gelly S. (2020). An image is worth 16x16 words: transformers for image recognition at scale. arXiv Prepr. arXiv2010.11929.

[18] Pantazopoulos G., Nikandrou M., Suglia A., Lemon O., Eshghi A. (2024).

[19] Zhang J., Huang J., Jin S., Lu S. (2023). Vision-language models for vision tasks: a survey. IEEE Trans. Pattern Anal. Mach. Intell..

[20] Devlin J., Cheng H., Fang H., Gupta S., Deng L., He X., Zweig G., Mitchell M. (2015). Language models for image captioning: the quirks and what works. ACL-IJCNLP 2015 - 53rd Annu. Meet. Assoc. Comput. Linguist. 7th Int. Jt. Conf. Nat. Lang. Process. Asian Fed. Nat. Lang. Process. Proc. Conf..

[21] Shi B., Bai X., Yao C. (2017). An end-to-end trainable neural network for image-based sequence recognition and its application to scene text recognition. IEEE Trans. Pattern Anal. Mach. Intell..

[22] Radford A., Kim J.W., Hallacy C., Ramesh A., Goh G., Agarwal S., Sastry G., Askell A., Mishkin P., Clark J. (February 26 2021). Proceedings of the International Conference on Machine Learning.

[23] Liu S., Zeng Z., Ren T., Li F., Zhang H., Yang J., Jiang Q., Li C., Yang J., Su H., Leonardis A., Ricci E., Roth S., Russakovsky O., Sattler T., Varol G. (2025). Grounding DINO: Marrying DINO with Grounded Pre-training for Open-Set Object Detection BT - Computer Vision – ECCV 2024.

[24] Li L., Li J., Chen D., Pu L., Yao H., Huang Y. (2025). *VLLFL: A Vision-Language Model Based Lightweight Federated Learning Framework for Smart Agriculture*. arXiv Prepr. arXiv:.

[25] Yu P., Lin B. (2024). A framework for agricultural intelligent analysis based on a visual language large model. Appl. Sci..

[26] Alayrac J.B., Donahue J., Luc P., Miech A., Barr I., Hasson Y., Lenc K., Mensch A., Millican K., Reynolds M. (2022). Flamingo: a visual language model for few-shot learning. Adv. Neural Inf. Process. Syst..

[27] Dai W., Li J., Li D., Tiong A.M.H., Zhao J., Wang W., Li B., Fung P., Hoi S. (2023).

[28] Yu W., Yang Z., Li L., Wang J., Lin K., Liu Z., Wang X., Wang L. (2024). Evaluating large multimodal models for integrated capabilities. Proc. Mach. Learn. Res..

[29] Bitton Y., Bansal H., Hessel J., Shao R., Zhu W., Awadalla A., Gardner J., Taori R., Schimdt, VisIT-Bench L. (2023). A benchmark for vision-language instruction following inspired by real-world use. Adv. Neural Inf. Process. Syst..

[30] Wang F., Fu X., Huang J.Y., Li Z., Liu Q., Liu X., Ma M.D., Xu N., Zhou W., Zhang K. (2024). MuirBench: a comprehensive benchmark for robust multi-image understanding. arXiv Prepr. arXiv2406.09411.

[31] Kpodo J., Kordjamshidi P., Nejadhashemi A.P. (2024). AgXQA: a benchmark for advanced agricultural extension question answering. Comput. Electron. Agric..

[32] Xiao B., Kantarci B., Kang J., Niyato D., Guizani M. (2024). Efficient prompting for LLM-based generative internet of things. IEEE Internet Things J..

[33] Javaid S., Fahim H., He B., Saeed N. (2024). Large language models for UAVs: current state and pathways to the future. IEEE Open J. Veh. Technol..

[34] Zhao B., Jin W., Ser Del, J, Yang G. (2023). ChatAgri: exploring potentials of ChatGPT on cross-linguistic agricultural text classification. Neurocomputing.

[35] Peng R., Liu K., Yang P., Yuan Z., Li S. (2023). Embedding-based retrieval with LLM for effective agriculture information extracting from unstructured data. arXiv Prepr. arXiv2308.03107.

[36] Zhu H., Qin S., Su M., Lin C., Li A., Gao J. (2024).

[37] Wang J. (August 29 2024). Proceedings of the International IEEE Conference Proceedings, IS.

[38] Wang X., Kim H., Rahman S., Mitra K., Miao Z. (2024). Proceedings of the Conference on Human Factors in Computing Systems - Proceedings.

[39] Selva Kumar S., Khan A.K.M.A., Banday I.A., Gada M., Shanbhag V.V. (2024). Proceedings of the International Conference on Emerging Technologies in Computer Science for Interdisciplinary Applications, ICETCS 2024.

[40] Singhal R., Izquierdo P., Ranaweera T., Segura Abá K., Brown B.N.I., Lehti-Shiu M.D., Shiu S.-H. (2025). Using supervised machine-learning approaches to understand abiotic stress tolerance and design resilient crops. Philos. Trans. R. Soc. B Biol. Sci..

[41] Murmu S., Sinha D., Chaurasia H., Sharma S., Das R., Jha G.K., Archak S. (2024). A review of artificial intelligence-assisted omics techniques in plant defense: current trends and future directions. Front. Plant Sci..

[42] Hunt C.H., Hayes B.J., van Eeuwijk F.A., Mace E.S., Jordan D.R. (2020). Multi-environment analysis of sorghum breeding trials using additive and dominance genomic relationships. Theor. Appl. Genet..

[43] Kushwaha S.K., Chauhan P., Hedlund K., Ahren D. (2016). NBSPred: a support vector machine-based high-throughput pipeline for plant resistance protein NBSLRR prediction. Bioinformatics.

[44] Ma C., Xin M., Feldmann K.A., Wang X. (2014). Machine learning-based differential network analysis: a study of stress-responsive transcriptomes in arabidopsis. Plant Cell.

[45] Mohanty S.P., Hughes D.P., Salathé M. (2016). Using deep learning for image-based plant disease detection. Front. Plant Sci..

[46] Joshi B.M., H. Bhavsar Deep Learning Technology Based Night-Cnn for Nightshade Crop Leaf Disease Detection (2023). Int. J. Intell. Syst. Appl. Eng..

[47] Islam M.M., Adil M.A.A., Talukder M.A., Ahamed M.K.U., Uddin M.A., Hasan M.K., Sharmin S., Rahman M.M., Debnath S.K. (2023). DeepCrop: deep learning-based crop disease prediction with web application. J. Agric. Food Res..

[48] Boukabouya R.A., Moussaoui A., Berrimi M. (November 29 2022). Proceedings of the ISIA 2022 - International Symposium on Informatics and Its Applications, Proceedings.

[49] Devlin J., Chang M.W., Lee K., Toutanova K. (2019). BERT: pre-training of deep bidirectional transformers for language understanding. Proceedings of the NAACL HLT 2019 - 2019 Conference of the North American Chapter of the Association for Computational Linguistics: Human Language Technologies - Proceedings of the Conference.

[50] Fedus W., Goodfellow I., Dai A.M. (2018). 6th Int. Conf. Learn. Represent. ICLR 2018 - Conf.

[51] Koh E., Sunil R.S., Lam H.Y.I., Mutwil M. (2024). Confronting the data deluge: how artificial intelligence can be used in the study of plant stress. Comput. Struct. Biotechnol. J..

[52] Li J., Li D., Savarese S., Hoi S. (2023). Proceedings of the Proceedings of the 40th International Conference on Machine Learning.

[53] Qing J., Deng X., Lan Y., Li Z. (2023). GPT-aided diagnosis on agricultural image based on a new light YOLOPC. Comput. Electron. Agric..

[54] Harfouche A.L., Jacobson D.A., Kainer D., Romero J.C., Harfouche A.H., Scarascia Mugnozza G., Moshelion M., Tuskan G.A., Keurentjes J.J.B., Altman A. (2019). Accelerating climate resilient plant breeding by applying next-generation artificial intelligence. Trends Biotechnol..

[55] De Clercq D., Nehring E., Mayne H., Mahdi A. (2024). Large language models can help boost food production, but be mindful of their risks. Front. Artif. Intell..

[56] Wang Y., Yao Q., Kwok J.T., Ni L.M. (2020). Generalizing from a few examples: a survey on few-shot learning. ACM Comput. Surv..

[57] Kavitha Lakshmi R., Savarimuthu N. (2021). Proceedings of the 2021 IEEE 6th International Conference on Computing, Communication and Automation.

[58] Abbas A., Jain S., Gour M., Vankudothu S. (2021). Tomato plant disease detection using transfer learning with C-GAN synthetic images. Comput. Electron. Agric..

[59] Feng L., Wu B., He Y., Zhang C. (2021). Hyperspectral imaging combined with deep transfer learning for rice disease detection. Front. Plant Sci..

[60] Zhang N., Wu H., Zhu H., Deng Y., Han X. (2022). Tomato disease classification and identification method based on multimodal fusion deep learning. Agric. For..

[61] Wang H., Shang S., Wang D., He X., Feng K., Zhu H. (2022). Plant disease detection and classification method based on the optimized lightweight YOLOv5 model. Agric. For..

[62] Wang T., Zhao L., Li B., Liu X., Xu W., Li J. (2022). Recognition and counting of typical apple pests based on deep learning. Ecol. Inform..

[63] Zhao Y., Sun C., Xu X., Chen J. (2022). RIC-Net: a plant disease classification model based on the fusion of inception and residual structure and embedded attention mechanism. Comput. Electron. Agric..

[64] Shin M.Y., Gonzalez Viejo C., Tongson E., Wiechel T., Taylor P.W.J., Fuentes S. (2023). Early detection of verticillium wilt of potatoes using near-infrared spectroscopy and machine learning modeling. Comput. Electron. Agric..

[65] Qadri S.A.A., Huang N.F., Wani T.M., Bhat S.A. (2023). Proceedings of the Proceedings - 13th IEEE International Conference on Control System, Computing and Engineering.

[66] Wang C., Zheng W., Zhu Z., Zhou J., Lu J. (2023). Proceedings of the Proceedings of the IEEE International Conference on Computer Vision.

[67] Sankareshwaran S.P., Jayaraman G., Muthukumar P., Krishnan A.S. (2023). Optimizing rice plant disease detection with crossover boosted artificial hummingbird algorithm based AX-RetinaNet. Environ. Monit. Assess..

[68] Catal Reis H., Turk V. (2024). Potato leaf disease detection with a novel deep learning model based on depthwise separable convolution and transformer networks. Eng. Appl. Artif. Intell..

[69] Malik M.E., Mahmud M.S. (2024). Proceedings of the 2024 ASABE Annual International Meeting.

[70] Zhang Y., Lv C. (2024). TinySegformer: a lightweight visual segmentation model for real-time agricultural Pest detection. Comput. Electron. Agric..

[71] Yang S., Yuan Z., Li S., Peng R., Liu K., Yang P. (2024). GPT-4 as evaluator: evaluating large language models on Pest management in agriculture. arXiv Prepr.

[72] Tzachor A., Devare M., Richards C., Pypers P., Ghosh A., Koo J., Johal S., King B. (2023). Large language models and agricultural extension services. Nat. Food.

[73] Wang T., Wang N., Cui Y., Liu J. (2023). Agricultural technology knowledge intelligent question-answering system based on large language model. Smart Agric.

[74] Chebbi A., Kniesel G., Abdennadher N., Dimarzo G. (2024). Proceedings of the EuroMLSys 2024 - Proceedings of the 2024 4th Workshop on Machine Learning and Systems.

[75] Rezayi S., Liu Z., Wu Z., Dhakal C., Ge B., Zhen C., Liu T., Li S. (July 2022). Proceedings of the Proceedings of the Thirty-First International Joint Conference on Artificial Intelligence.

[76] Ray P.P. (2023). AI-Assisted sustainable farming: harnessing the power of ChatGPT in modern agricultural sciences and technology. ACS Agric. Sci. Technol..

[77] Nismi Mol E.A., Santosh Kumar M.B. (2024). End-to-End framework for agricultural entity extraction – a hybrid model with transformer. Comput. Electron. Agric..

[78] Mendoza-Revilla J., Trop E., Gonzalez L., Roller M., Dalla-Torre H., de Almeida B.P., Richard G., Caton J., Lopez Carranza N., Skwark M. (2024). A foundational large language model for edible plant genomes. Commun. Biol..

[79] Sapkota R., Qureshi R., Hassan S.Z., Shutske J., Shoman M., Sajjad M., Dharejo F.A., Paudel A., Li J., Meng Z. (2024).

[80] Cao Y., Chen L., Yuan Y., Sun G. (2023). Cucumber disease recognition with small samples using image-text-label-based multi-modal language model. Comput. Electron. Agric..

[81] Madaan V., Bindal G., Singh S., Yadav S.K., Singh A., Sinha P., Nagpal D. (2024). Integrating language models and machine learning for crop disease detection for farmer guidance. Proceedings of the CEUR Workshop Proceedings.

[82] Arshad M.A., Jubery T.Z., Roy T., Nassiri R., Singh A.K., Singh A., Hegde C., Ganapathysubramanian B., Balu A., Krishnamurthy A. (2024). AgEval: a benchmark for zero-shot and few-shot plant stress phenotyping with multimodal LLMs.

[83] Lu Y., Lu X., Zheng L., Sun M., Chen S., Chen B., Wang T., Yang J., Lv C. (2024). Application of multimodal transformer model in intelligent agricultural disease detection and question-answering systems. Plants.

[84] Hue Y., Kim J.H., Lee G., Choi B., Sim H., Jeon J., Ahn M. Il, Han Y.K., Kim K.T. (2024). Artificial intelligence plant doctor: plant disease diagnosis using GPT4-Vision. Res. Plant Dis..

[85] Dhavale C., Pawar T., Singh A., Pole S., Sabat K. (February 8 2024). Proceedings of the 2024 2nd International Conference on Computer, Communication and Control.

[86] Chen F., Tsaftaris S.A., Giuffrida V. (August 13 2024). Proceedings of the the 34th European Conference on Computer Vision.

[87] Madhavi, Singh T.P., Singh S.P. (2025). DeepLabV3+ with GPT: a framework for leaf disease image to text generation. Procedia Comput. Sci..

[88] Zhu H., Shi W., Guo X., Lyu S., Yang R., Han Z. (2025). Potato disease detection and prevention using multimodal AI and large language model. Comput. Electron. Agric..

[89] Zhao X., Chen B., Ji M., Wang X., Yan Y., Zhang J., Liu S., Ye M., Lv C. (2024). Implementation of large language models and agricultural knowledge graphs for efficient plant disease detection. Agric. For..

[90] Slimani H., Mhamdi J. El, Jilbab A. (2023). Drone-assisted plant disease identification using artificial intelligence: a critical review. Int. J. Comput. Digit. Syst..

[91] Zhang Z., Amiri H., Liu Z., Züfle A., Zhao L. (October 29 2023). Proceedings of the Proceedings of the 1st ACM SIGSPATIAL International Workshop on Geospatial Anomaly Detection.

[92] Yang J., Liu C., Deng W., Wu D., Weng C., Zhou Y., Wang K. (2024). Enhancing phenotype recognition in clinical notes using large language models: PhenoBCBERT and PhenoGPT. Patterns.

[93] Khey H., Lakhder A., Rouichi S., Ghabi I. El, Hejjaoui K., En-nahli Y., Kalloubi F., Amri M. (2025).

[94] Dofitas C., Kim Y.W., Byun Y.C. (2025). Advanced agricultural query resolution using ensemble-based large language models. IEEE Access.

[95] Nam D., MacVean A., Hellendoorn V., Vasilescu B., Myers B. (2024). Proceedings of the Proceedings - International Conference on Software Engineering.

[96] Gao M., Hu X., Ruan J., Pu X., Wan X. (2024). Llm-based Nlg evaluation: current status and challenges. arXiv Prepr. arXiv2402.01383.

[97] Shutske J.M. (2023). Editorial: harnessing the power of large language models in agricultural safety & health. J. Agric. Saf. Health.

[98] Panoutsopoulos H., Espejo-Garcia B., Raaijmakers S., Wang X., Fountas S., Brewster C. (2024). Investigating the effect of different fine-tuning configuration scenarios on agricultural term extraction using BERT. Comput. Electron. Agric..

[99] Fang X., Xu W., Tan F.A., Zhang J., Hu Z., Qi Y., Nickleach S., Socolinsky D., Sengamedu S., Faloutsos C. (2024). Large language Models(LLMs) on tabular data: prediction, Generation, and Understanding -- A Survey. arXiv Prepr.

[100] Weichelt B.P., Pilz M., Burke R., Puthoff D., Namkoong K. (2024). The potential of AI and ChatGPT in improving agricultural injury and illness surveillance programming and dissemination. J. Agromed..

[101] Abbas A., Jain S., Gour M., Vankudothu S. (2021). Tomato plant disease detection using transfer learning with C-GAN synthetic images. Comput. Electron. Agric..

[102] Pan K., Zhang X., Chen L. (2024). Research on the training and application methods of a lightweight agricultural domain-specific large language model supporting mandarin Chinese and Uyghur. Appl. Sci..

[103] Huang L., Yu W., Ma W., Zhong W., Feng Z., Wang H., Chen Q., Peng W., Feng X., Qin B. (2023). A survey on hallucination in large language models: principles, taxonomy, challenges, and open questions. ACM Trans. Inf. Syst..

[104] Guu K., Lee K., Tung Z., Pasupat P., Chang M. (2020). Proceedings of the International Conference on Machine Learning.

[105] Renze M., Guven E. (2024). Proceedings of the Findings of the Association for Computational Linguistics: EMNLP 2024.

[106] Karmaker S.K., Feng D. (2023 2023). TELeR: a general taxonomy of LLM prompts for benchmarking complex tasks. Find. Assoc. Comput. Linguist. EMNLP.

[107] French R.M. (1999). Catastrophic forgetting in connectionist networks. Trends Cognit. Sci..

[108] Yang G., Pan F., Gan W.B. (2009). Stably maintained dendritic spines are associated with lifelong memories. Nature.

[109] Kirkpatrick J., Pascanu R., Rabinowitz N., Veness J., Desjardins G., Rusu A.A., Milan K., Quan J., Ramalho T., Grabska-Barwinska A. (2017). Overcoming catastrophic forgetting in neural networks. Proc. Natl. Acad. Sci. U. S. A.

[110] Mahapatra J., Garain U. (2024). Impact of model size on fine-tuned LLM performance in data-to-text generation: a state-of-the-art investigation. arXiv Prepr. arXiv2407.14088.

[111] Yadav S., Kaushik A., Sharma M., Sharma S. (2022). Disruptive technologies in smart farming: an expanded view with sentiment analysis. AgriEngineering.

[112] Arshad M.A., Jubery T.Z., Roy T., Nassiri R., Singh A.K., Singh A., Hegde C., Ganapathysubramanian B., Balu A., Krishnamurthy A. (2025). Leveraging vision language models for specialized agricultural tasks.

[113] Nawaz U., Awais M., Gani H., Naseer M., Khan F., Khan S., Anwer R. (2024).

[114] Gauba A., Pi I., Man Y., Pang Z., Adve V.S., Wang Y.-X. (2025).

[115] Awais M., Salem Abdulla Alharthi A.H., Kumar A., Cholakkal H., Anwer R.M. (2025 2024). AgroGPT: efficient agricultural vision-language model with expert tuning. Proc. - 2025 IEEE Winter Conf. Appl. Comput. Vision, WACV.

[116] Yang X., Gao J., Xue W., Alexandersson E. (2024).

[117] Szilágyi R., Tóth M. (2024). Use of LLM for SMEs, opportunities and challenges. J. Agric. Informatics.

[118] Ali M., Fromm M., Thellmann K., Rutmann R., Lübbering M., Leveling J., Klug K., Ebert J., Doll N., Buschhoff J.S. (2024). Proceedings of the Findings of the Association for Computational Linguistics: NAACL 2024 - Findings.

[119] Cagnetti C., Braccini A.M. (2023). Business information systems adoption in agriculture 4.0: a sociotechnical exploration of enabling factors, barriers, and outcomes. Proceedings of the CEUR Workshop Proceedings.

[120] Adli H.K., Remli M.A., Wan Salihin Wong K.N.S., Ismail N.A., González-Briones A., Corchado J.M., Mohamad M.S. (2023). Recent advancements and challenges of AIoT application in smart agriculture: a review. Sensors.

[121] Musafiri C.M., Kiboi M., Macharia J., Ng’etich O.K., Kosgei D.K., Mulianga B., Okoti M., Ngetich F.K. (2022). Adoption of climate-smart agricultural practices among smallholder farmers in Western Kenya: do socioeconomic, institutional, and biophysical factors matter?. Heliyon.

[122] Ndlovu B., Dube S., Maguraushe K., Princess Dube S. (July 16 2024). Proceedings of the Proceedings of the International Conference on Industrial Engineering and Operations Management.

[123] Kabir K., de Vries Robbe S., Godinho C. (2024). Climate change mitigation policies in agriculture: an overview of sociopolitical barriers. Wiley Interdiscip. Rev. Clim. Chang..

[124] Kuska M.T., Wahabzada M., Paulus S. (2024). AI for crop production – where can large language models (LLMs) provide substantial value?. Comput. Electron. Agric..

[125] Xu Y., Lu J., Zhang J. (2024 2024). Bridging the gap between different vocabularies for LLM ensemble. Proc. 2024 Conf. North Am. Chapter Assoc. Comput. Linguist. Hum. Lang. Technol. NAACL.

[126] Sun Y., Ortiz J. (2024). An AI-Based system utilizing IoT-Enabled ambient sensors and LLMs for complex activity tracking. Acad. J. Sci. Technol..

[127] Kaur J., Hazrati Fard S.M., Amiri-Zarandi M., Dara R. (2022). Protecting farmers' data privacy and confidentiality: recommendations and considerations. Front. Sustain. Food Syst..

[128] Wang Y., Feng D., Dai Y., Chen Z., Huang J., Ananiadou S., Xie Q., Wang H. (2024). HARMONIC: harnessing LLMs for tabular data synthesis and privacy protection. arXiv Prepr. arXiv2408.02927.

[129] Jayasinghe, U.; Ghoshal, D. Fertiliser Ban Decimates Sri Lankan Crops as Government Popularity Ebbs. Https://Www.Reuters.Com/Markets/Commodities/Fertiliser-Ban-Decimates-Sri-Lankan-Crops-Government-Popularity-Ebbs-2022-03-03/2023, 3.

[130] Dong X., Wang Y., Yu P.S., Caverlee J. (2023). Probing explicit and implicit gender bias through LLM conditional text generation. arXiv Prepr. arXiv2311.00306.

[131] Peng B., Bi Z., Niu Q., Liu M., Feng P., Wang T., Yan L.K.Q., Wen Y., Zhang Y., Yin C.H. (2024). Jailbreaking and mitigation of vulnerabilities in large language models. arXiv Prepr. arXiv2410.15236.

[132] Peng B., Chen K., Li M., Feng P., Bi Z., Liu J., Niu Q. (2024). Securing large language models: addressing bias, misinformation, and prompt attacks. arXiv Prepr. arXiv:2409.08087.

[133] Das B.C., Amini M.H., Wu Y. (2024). Security and privacy challenges of large language models: a survey. ArXiv.

[134] Tang X., Jin Q., Zhu K., Yuan T., Zhang Y., Zhou W., Qu M., Zhao Y., Tang J., Zhang Z. (2025). Risks of AI Scientists: Prioritizing Safeguarding over Autonomy. Nat. Commun..

[135] Zhang Y., Yao S., Wang P., Wu H., Xu Z., Wang Y., Zhang Y. (2022). Building natural language interfaces using natural language understanding and generation: a case study on human–machine interaction in agriculture. Appl. Sci..

[136] Jayasingh D.K., Anand A., Das K.S., Anshuman J., Mallick B. (2023). Innovative Agriculture: Strategies and Concepts in Extension.

[137] Palma R., Roussaki I., Döhmen T., Atkinson R., Brahma S., Lange C., Routis G., Plociennik M., Mueller S. (2022). Springer Optimization and its Applications.

[138] Veena G., Kanjirangat V., Gupta D. (2023). AGRONER: an unsupervised agriculture named entity recognition using weighted distributional semantic model. Expert Syst. Appl..

[139] Ali S., Abuhmed T., El-Sappagh S., Muhammad K., Alonso-Moral J.M., Confalonieri R., Guidotti R., Del Ser J., Díaz-Rodríguez N., Herrera F. (2023). Explainable artificial intelligence (XAI): what we know and what is left to attain trustworthy artificial intelligence. Inf. Fusion.

[140] Williamson H.F., Brettschneider J., Caccamo M., Davey R.P., Goble C., Kersey P.J., May S., Morris R.J., Ostler R., Pridmore T. (2023). Data management challenges for artificial intelligence in plant and agricultural research. F1000Research.

[141] Saranya A., Subhashini R. (2023). A systematic review of explainable artificial intelligence models and applications: recent developments and future trends. Decis. Anal. J..

[142] Linardatos P., Papastefanopoulos V., Kotsiantis S. (2021). Explainable ai: a review of machine learning interpretability methods. Entropy.

[143] Szegedy C., Zaremba W., Sutskever I., Bruna J., Erhan D., Goodfellow I., Fergus R. (2014). 2nd Int. Conf. Learn. Represent. ICLR 2014 - Conf.

[144] Abburi H., Suesserman M., Pudota N., Veeramani B., Bowen E., Bhattacharya S. (2023).

[145] Schoenegger P., Tuminauskaite I., Park P.S., Bastos R.V.S., Tetlock P.E. (2024). Wisdom of the silicon crowd: LLM ensemble prediction capabilities rival human crowd accuracy. Sci. Adv..

[146] Gregor H.F. (1982).

[147] Nagasubramanian G., Sakthivel R.K., Patan R., Sankayya M., Daneshmand M., Gandomi A.H. (2021). Ensemble classification and IoT-Based pattern recognition for crop disease monitoring system. IEEE Internet Things J..

[148] Tao Z., Wei Y., Wang X., He X., Huang X., Chua T.S. (2020). MGAT: multimodal graph attention network for recommendation. Inf. Process. Manag..

[149] Zhai Z., Martínez J.F., Beltran V., Martínez N.L. (2020). Decision support systems for agriculture 4.0: survey and challenges. Comput. Electron. Agric..

[150] Inoue Y. (2020). Satellite- and drone-based remote sensing of crops and soils for smart farming – a review. Soil Sci. Plant Nutr..

[151] Cai K., Duan Z., Liu G., Fleming C., Lu C.X. (2025).

[152] de Curtò J., de Zarzà I., Calafate C.T. (2023). Semantic scene understanding with large language models on unmanned aerial vehicles. Drones.

[153] Rose C., McMurray R., Hadi M.U. (2024). Proceedings of the Proceedings of the 35th Irish Systems and Signals Conference, ISSC 2024.

[154] Rajathanakodi R., Hadi M.U. (2024). Proceedings of the Proceedings of the 35th Irish Systems and Signals Conference, ISSC 2024.

[155] Singh N., Gupta D., Joshi M., Yadav K., Nayak S., Kumar M., Nayak K., Gulaiya S., Rajpoot A.S. (2024). Application of drones technology in agriculture: a modern approach. J. Sci. Res. Reports.

[156] Sun L., Qin M., Jin Y., Peng B. (2024). From bytes to bites: large language models revolutionizing agriculture. preprint.

[157] Yu Z., Wu Y., Zhang N., Wang C., Vorobeychik Y., Xiao C. (2023). Proceedings of the Proceedings of Machine Learning Research.

[158] Nguyen T., Van Nguyen C., Lai V.D., Man H., Ngo N.T., Dernoncourt F., Rossi R.A., Nguyen T.H. (2024). CulturaX: a cleaned, enormous, and multilingual dataset for large language models in 167 languages. Jt. Int. Conf. Comput. Linguist. Lang. Resour. Eval. Lr. 2024 - Main Conf. Proc..

[159] Alam F., Chowdhury S.A., Boughorbel S., Hasanain M. (2024). Proceedings of the EACL 2024 - 18th Conference of the European Chapter of the Association for Computational Linguistics, Proceedings of Tutorial Abstracts.

[160] Shukla N.K., Katikeri R., Raja M., Sivam G., Yadav S., Vaid A., Prabhakararao S. (2023). Proceedings of the Proceedings - 2023 IEEE International Conference on Big Data, BigData 2023.

[161] Lankford S., Way A. (2024). Leveraging LLMs for MT in crisis scenarios: a blueprint for low-resource languages. AMTA 2024 - 16th Conf. Assoc. Mach. Transl. Am..

[162] Sun L., Qin M., Peng B. (2024).

[163] Chakka G.S.V.C., Aridoss M., Jayapal S. (2024). From bytes to bites: revolutionizing culinary creativity through deep learning and AI-Generated recipes. Studies in Computational Intelligence.

